# The Mechanistic Basis of *Myxococcus xanthus* Rippling Behavior and Its Physiological Role during Predation

**DOI:** 10.1371/journal.pcbi.1002715

**Published:** 2012-09-27

**Authors:** Haiyang Zhang, Zalman Vaksman, Douglas B. Litwin, Peng Shi, Heidi B. Kaplan, Oleg A. Igoshin

**Affiliations:** 1 Department of Bioengineering, Rice University, Houston, Texas, United States of America; 2 Department of Microbiology and Molecular Genetics, University of Texas Medical School, Houston, Texas, United States of America; University of Notre Dame, United States of America

## Abstract

*Myxococcus xanthus* cells self-organize into periodic bands of traveling waves, termed ripples, during multicellular fruiting body development and predation on other bacteria. To investigate the mechanistic basis of rippling behavior and its physiological role during predation by this Gram-negative soil bacterium, we have used an approach that combines mathematical modeling with experimental observations. Specifically, we developed an agent-based model (ABM) to simulate rippling behavior that employs a new signaling mechanism to trigger cellular reversals. The ABM has demonstrated that three ingredients are sufficient to generate rippling behavior: (i) side-to-side signaling between two cells that causes one of the cells to reverse, (ii) a minimal refractory time period after each reversal during which cells cannot reverse again, and (iii) physical interactions that cause the cells to locally align. To explain why rippling behavior appears as a consequence of the presence of prey, we postulate that prey-associated macromolecules indirectly induce ripples by stimulating side-to-side contact-mediated signaling. In parallel to the simulations, *M. xanthus* predatory rippling behavior was experimentally observed and analyzed using time-lapse microscopy. A formalized relationship between the wavelength, reversal time, and cell velocity has been predicted by the simulations and confirmed by the experimental data. Furthermore, the results suggest that the physiological role of rippling behavior during *M. xanthus* predation is to increase the rate of spreading over prey cells due to increased side-to-side contact-mediated signaling and to allow predatory cells to remain on the prey longer as a result of more periodic cell motility.

## Author Summary

*Myxococcus xanthus* cells collectively move on solid surfaces and reorganize their colonies in response to environmental cues. Under some conditions, cells exhibit an intriguing form of collective motility by self-organizing into bands of travelling alternating-density waves termed ripples. These waves are distinct from the waves originating from Turing instability in diffusion-reaction systems, as these counter-traveling waves do not annihilate but appear to pass through each other. Here we developed a new mathematical model of rippling behavior based on a recently observed contact signaling mechanism – cells that make side-to-side contacts can signal one another to reverse. We hypothesize that this signaling is enhanced by the presence of prey-associated macromolecules and compare modeling predictions with experimentally observed waves generated on *E. coli* prey cells. The model predicts a modified relationship between the wavelength and individual predatory cell motility parameters and provides a physiological role for rippling during predation. We show that ripples allow predatory cells to increase the rate of their spreading to quickly envelope the prey, and subsequently to decrease their random drift to remain in the prey region for longer. These and other predictions are confirmed by the experimental observations.

## Introduction

Spatial self-organization of developing cells, which results the formation of complex dynamic structures, remains one of the most intriguing phenomena in modern biology [1]–[4]. Analogous developmental behaviors are observed as bacterial cells form biofilms, which are populations of surface-associated cells enclosed in a self-produced matrix [5], [6]. The dynamic self-organization in biofilms formed by the soil bacterium *Myxococcus xanthus* is dependent on the ability of the cells to move on solid surfaces [7], [8], while sensing, integrating and responding to a variety of intercellular and environmental cues [9]–[12].

*M. xanthus* is the preeminent model system for bacterial social development. At high density and under nutrient stress *M. xanthus* cells execute a complex multicellular developmental program by aggregating into multicellular mounds, termed fruiting bodies, and differentiating into dormant, environmentally resistant myxospores [11]. In addition, these bacteria exhibit complex behaviors when they cooperatively prey on other microorganisms by collectively spreading over the prey cells, producing antibiotics and lytic compounds that kill and decompose their prey [13], [14]. One of the most intriguing forms of collective dynamics exhibited by *M. xanthus* is their ability to self-organize into ripples – travelling bands of high-density wave crests [15]–[18]. Although the *M. xanthus* counter-traveling waves appear to pass through each another, they actually reflect off of one another and are termed “accordion waves” [16], [18]–[21]. These waves are distinct from the waves originating from Turing instability diffusion-reaction patterns, such as those in chemical systems or observed during development of the other well-studied model social microorganism, the amoeba *Dictyostelium discoideum* [22]–[24].

The initial studies of the mechanisms underlying *M. xanthus* rippling motility focused on this behavior during starvation-induced multicellular fruiting body development [16]–[20], [25]–[27]. The application of mathematical modeling to developmental rippling revealed that the wave properties are consistent with contact-induced reversal signaling [18]–[21], [28]. This signaling was hypothesized to originate from ‘head-to-head’ collisions of cells moving in opposite directions and to result in an exchange of C-signal that accelerates the reversal clock [16], [19], [20]. C-signal is an extracellular protein that controls aggregation and sporulation via contact-dependent pole-to-pole transmission [12]. Developmental aggregation and motility coordination are induced through the C-signal-dependent stimulation of the *frz* chemotaxis-like system, which includes an unconventional soluble cytoplasmic chemoreceptor homologue FrzCD [12], [29], [30].

An opportunity to reevaluate and replace the pole-to-pole collision-mediated model was prompted by a new report of FrzCD protein clusters that appear to transiently align and stimulate reversals in cells making side-to-side contact [8] and by the recent discovery that more robust rippling occurs during predation [13], [15]. In this paper we have investigated predatory rippling behavior with a combination of mathematical modeling and experimentation. We have constructed a mathematical model that faithfully reproduces the travelling wave behavior by adapting the recently proposed reversal-inducing side-to-side contact-mediated signaling model [8] and incorporating the properties of the patterns resulting from these interactions.

## Results

### A new agent-based model reproduces rippling self-organization

To model collective cell behavior we needed a modeling formalism that would allow us to connect the motility of individual cells, intercellular interactions, and the resulting population patterns. To this end, we employed an agent-based model (ABM) approach [19], [31]–[33]. Individual cells are represented as agents that move and interact according to the rules and equations that correspond to experimental observations. Unlike continuous, cell-density-based approaches, the ABM approach allows cell variability and modular implementation of interactions to be easily incorporated. The details and equations describing our ABM are summarized in the Materials and Methods Section. Here we qualitatively describe the main model ingredients that result in predatory rippling behavior.

Each agent is simulated as a self-propelled rod on a 2-D surface. The agents move continuously along their long axis and periodically reverse by switching the polarity of their two ends simultaneously. As in the previous models [19]–[21], we expected the ripples to emerge as a result of intercellular signaling, which leads to synchronized cellular reversals among the cell population. The side-to-side contact-induced signaling mechanism used here is based on the recent observations by Mauriello et al. [8], which demonstrated that when cells make transient side-to-side contact, their FrzCD clusters align causing one or both of the cells to reverse. The reversals stimulated by this intercellular signaling would be somewhat similar to the reversals induced by pole-to-pole collisions that were hypothesized to occur due to C-signal exchange during *M. xanthus* development [18]–[21]. Based on this and other experimental observations, our model incorporates four rules to guide the agents' interactions (see below). These rules are converted to mathematical equations that describe rippling motility (see the Materials and Methods Section).

- Two counter-moving agents that make side-to-side contact with a minimal length overlap (Figure S1) have a probability of engaging in a signaling event that results in the reversal of at least one of the agents.
- Agents enter a refractory period after each reversal during which another reversal will not occur.
- Agents align locally along their long axes as a result of their physical interactions.
- Agents without side-to-side contact spontaneously reverse with a mean period about three times greater than the mean refractory period.

**Figure 1:**
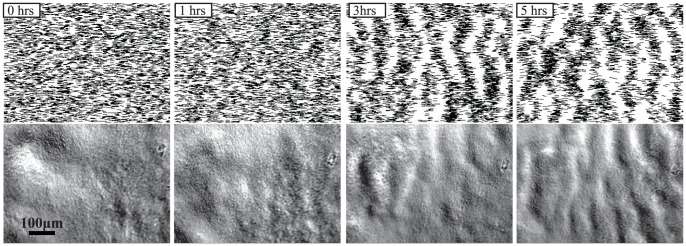
Comparison of ripple initiation in the ABM simulations (top panels) and experiments (bottom panels). The timing of the snapshot is indicated for each column. The initial time (0 hrs) corresponds to the initiation of the simulation with a uniform cell distribution or the time *M. xanthus* cells fully cover the prey in the field of view. The fields of view of both the ABM simulation images and experimental images have the same dimensions; the scale bar is 100 µm.

The first three rules are sufficient for the model to produce rippling behavior (Figure 1, top row; Video S1). Starting from a uniform aligned population of agents (0 hrs), the model results in their self-organization into periodic traveling bands (ripples) within about 3 hrs. As in previous models [18]–[21], [25]–[27], the ripples emerge from the synchronized reversals. However, this model, which is based on a side-to-side contact-mediated signaling mechanism, appears to be more robust than the previous models that utilized pole-to-pole collision-mediated signaling (Figure S2). Rule (iv) is not necessary for rippling, but it allows the model to reflect the cell reversal behavior exhibited at low densities when cell contacts are rare, and it does not significantly change the high-density motility patterns studies here. The mean value of the native reversal period is chosen to be about 8 min (Figure S3 A) to achieve agreement with experimental observations by us here and others [11].

Within the framework of the proposed model, each rule (i)–(iii) is necessary to generate rippling behavior. Specifically, rule (i) is necessary because eleminating intercellular reversal-generated signaling abolishes rippling motility (data not shown) and eliminating the assumptions that signaling occurs only between counter-moving agents has the same effect (Figure S4 A and B). It is noteworthy that rippling motility is robust to the minimal overlap between agents that is required for them to engage in side-to-side signaling (Figure S5). Hereafter, an arbitrary value of 50% as a minimal overlap threshold is assumed in all simulations. Moreover, Figure S4 C vs D show that rippling motility occurs regardless of whether each signaling event is bidirectional (when cell #1 signals to cell #2, cell #2 also signals to cell #1) or unidirectional (cell #1 signaling to cell #2 and cell #2 signaling to cell #1 are independent events). In our simulations we use unidirectional signaling assumptions for the reasons explained below. The refractory period (rule ii) is also required for ripples, as reducing it to a very short duration leads to the dissapearance of the waves (Figure S4 E and F). In our simulations, the refractory period is a stochastic quantity with a mean value of 2.7 min and standard deviation of 0.7 min (Figure S3 B). Side-to-side signaling and rippling motility can only occur in a locally aligned cell population, and thus, physical interaction aligning cells, rule (iii), is necesary to maintain the cells' long axes approximately parallel.

Since in our simulations the rules (i)–(iii) induce rippling motility, we addressed the question of which rule is modulated to ensure that rippling motility is observed only when prey cells or the macromolecules associated with their lysis are present. The initiation and maintance of ripples seems to depend on the probability of reversal-inducing signaling events (Figure S6), which must exceed a threshhold value of ∼5–10%. If the probability is below 5%, then the ripples will not form and the agents will remain uniformly distributed on the 2-D surface. When the signaling probability exceeds the threshold value, the uniform distribution becomes unstable and the agents self-organize into ripples. Therefore, we hypothesize that the presence of prey-associated macromolecules indirectly stimulates rippling by increasing the probability that side-to-side contact generates successive signaling events (reversals). Although the biochemical mechanism of this induction is unknown, various macromolecular substrates, such as peptidoglycan, bovine serum albumin, and salmon testes chromosomal DNA, have been shown to induce rippling motility [13], [15]. Thus, we predict that the presence of these substrates should increase the probability of reversal-inducing signaling. Although our experimental arrangement does not allow direct testing of this prediction, we can quantitatively compare the emergent properities of the rippling patterns in the model and in the experiments.

It should be noted that the experiments demonstrating side-to-side signaling were preformed in the absence of prey cells or prey-associated macromolecules [8]. However, the results reported by Mauriello et al. [8] are consistent with a low probability of side-to-side signaling and the assumption that signaling is unidirectional. This is because in their observations only one of the cells engaged in side-to-side contact signaling reverses its gliding direction [8] (see also Figure S7). If the probability of signaling is low, it is unlikely that two signaling events will occur simultaneously. Furthermore, once one of the cell reverses, both cells will then be moving in the same direction and as a result, they will no longer be capable of signaling one another.

### Quantifying individual and collective cell behavior in predatory ripples with fluorescence microscopy

To test the modeling predictions experimentally, we observed cell motility on a solid nutrient agar surface in the presence of prey cells. The ripples were observed with fluorescence and differential interference contrast (DIC) time-lapse microscopy, allowing us to track cell density changes and the motility of a small percentage (0.5%) of GFP expressing cells in a wild-type population (see Materials and Methods section and Video S2). These images allowed us to calculate the global properties of the ripples: wavelength (distance from one wave crest to the next) and wave-crest width, and at the same time to measure the behavioral properties of individual cells: coordinates, velocity, reversal period, and the time/position of cellular reversals.

These data provided crucial input into the model and allowed us to test our modeling predictions. It is clear that the experimental ripple patterns appear very similar to those produced in the simulation (Figure 1). To compare the timing of wave initiation between the mathematical model and the experimental results, the time point when *M. xanthus* cells fully cover the prey in the field of view was chosen as the starting time (0 hrs in Figure 1; Video S3). Snapshot images at 0, 1, 3 and 5 hrs were selected to show the process of ripple formation in both systems. The experimental process of wave initiation appears to follow the same dynamics as the simulations. Initially, the cells homogeneously cover the field of view and the cells align as they cover the prey. During the first 3 hrs the reversals of individual cells become synchronous and result in the formation of ripples. By 5 hrs the ripples are pronounced and are easily discernible.

These results indicate that the ABM is capable of qualitatively reproducing the dynamics of rippling motility observed under our experimental conditions. Interestingly, waves generated with the ABM appear somewhat more pronounced than experimentally observed ripples, which have a smaller cell density gradient from crest to trough. This observation suggests that not all the cells in the biofilm participate in rippling behavior.

### Wavelength quantification is consistent with the proposed rippling mechanism

To compare the rippling patterns produced by the ABM to those of the experiments, we quantitatively characterized the ripples and related their patterns to the behavior of individual cells. Previous models of rippling motility [20], [21] proposed a simple equation, which relates wavelength (λ), individual agent speed (*v*), and agent reversal period (τ):
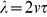
(1)This equation indicates that cells in two colliding crests (relative speed 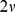) reverse their directions every time the crests are superimposed. This prediction was confirmed by both the ABM and experimental results of developing cells [19]. However, our analysis of the measurements by Berleman et al. [13], showed that wavelengths of their predatory ripples were ∼50% larger than those predicted by Eq. (1). Using their experimental values of *v* = 3 µm/min and *τ* = 8 minutes, the calculated λ should be 48 µm, however their observed λ was ∼70 µm.

**Figure 2:**
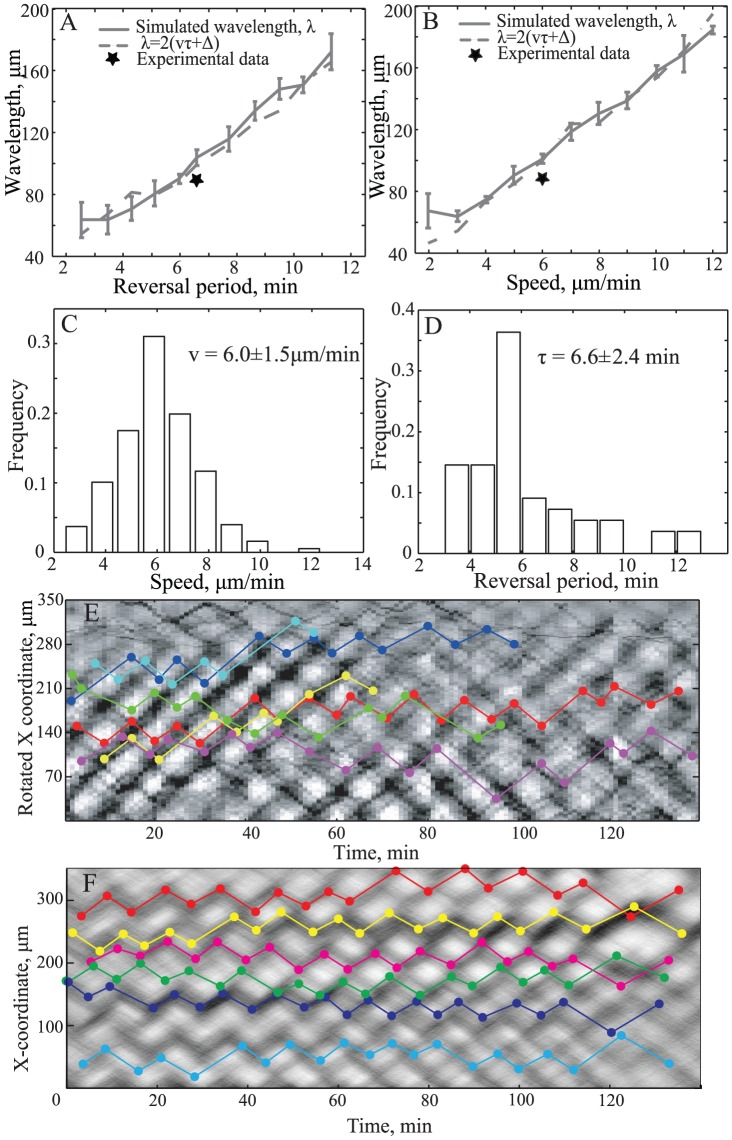
The relationship between the wavelength and individual cell motility. The ABM simulations show that the wavelength linearly scales with (A) a varying average reversal period and (B) a varying cell velocity. The velocity predicted by Eq. (2) is shown by a dashed line and is in good agreement with the wavelength calculated from ABM simulations (solid line – mean values; error bars – standard deviations). The experimentally measured wavelength (stars) also agrees with the ABM predictions based on the average values computed from the measured distributions of speed (C) and reversal period (D). The distributions are obtained from tracking fluorescently-labeled cells in microscopic images. (E) Superposing the trajectories of six cells on a space-time plot (colored lines) of 1-D averaged intensity images of ripples experimentally confirms that most cell reversals (colored dots) occur when two wave crests collide. (F) Same as E but using the ABM data. The reversals of the agents usually occur during wave crest collisions (92%±1.2%), as all of the cells participate in rippling. For comparison, we would expect about 17% (±2.3%) of the reversals to occur in wave crests in a hypothetical control population that does not sense side-to-side contact-mediated signaling.

To determine if the wavelength relationship, Eq. (1), works for our new ABM of rippling motility, two sets of simulations were conducted. First, the agent speed was fixed at 6 µm/min, while the spontaneous reversal period was varied between 5 min and 30 min (corresponding to the variation between 3 and 12 min of an actual average reversal period, which is smaller due to early reversals triggered by side-to-side contact signaling; Figure 2A, solid line). Second, the spontaneous reversal period was fixed at a value corresponding to an average reversal period of approximately 6.6 min and the cell speed was varied between 2 µm/min and 12 µm/min (Figure 2B, solid line). These fixed values correspond to the experimental cell motility parameters. As shown in Figure 2A and 2B, the wavelength (*λ*) scales linearly with agent speed (*v*) and average reversal period (*τ*). However, when no-intercept linear regression was used, regression coefficients of 15.2 µm/min for Figure 2A and 16.1 min for Figure 2B were obtained. Both values are slightly larger than the predicted coefficients of 2*v* (12 µm/min) and 2*τ* (13.2 min), respectively. When we tracked the reversal points of individual agents, we observed that the agent reversals were initiated as soon as the leading edge of each crest came into contact (Test S1; Figure S6). This indicates that as the agents at the front of each crest reverse, they signal to the other cells in their crests, leading to a “chain-reaction” of signaling and reversals. Given the wave crest width Δ, the cells in each crest only move an average distance of λ−2Δ before reversing again, which results in the average reversal period τ = (λ−2Δ)/2v. Thus, we modified our wavelength equation to be:
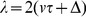
(2)To test the modified expression in our simulations, we automatically computed the average wave-crest width Δ from the simulation results (see Text S1) and used it to compute the wavelength with Eq. (2). The results demonstrate good agreement between the simulated and predicted wavelengths (Figure 2 A and B, solid vs. dashed line).

To test the Eq. (2) prediction experimentally for predatory rippling motility, we tracked 37 GFP-labeled individual cells within ripples for about 2 hr (or until the cells left the field of view). Continuous 1-D wavelet transform of the microscopy images (see Text S1) was used to compute the wavelength and wave-crest width by fitting a Gaussian function to the wave crest calculations. The distributions of average speed and reversal period are shown in Figures 2 C and D; and the ABM-predicted wavelengths are in agreement with the experimentally observed wavelength (denoted by the stars in Figures 2A and B). The prediction of Eq. (2) is also in good agreement with the data from Berleman et al. [13]. Using their experimentally derived values of *v* = 3 µm/min, *τ* = 8 min, *Δ*∼10–15 µm, the wavelength, *λ*, is calculated at ∼70–80 µm, which matches their published values. Rippling motility simulated with these parameters is shown in Video S4. To further test modeling predictions, we attempted to alter rippling wavelengths with changes in agar density and initial prey-cell concentration. We have selected two plates displaying reduced wavelength for detailed analysis and cell tracking. The results show that predictions of Eq. (2) also hold for these data: a reduced wavelength resulted from a reduction in the cell speed in both movies (∼3 µm/min) and a reduction of the reversal frequency (∼4.5 min) in one of the movies. Table S2 summarizes our experimental tests of Eq. (2).

According to our ABM assumptions and predictions, most of the rippling cells should travel with the wave crest and reverse, essentially as a group, when the leading edges of the two opposing wave crests collide. To test this prediction, we observed reversals of individual cells in the context of wave-crest movement by plotting cell trajectories on the space-time florescence intensity of ripples (Figure 2E). The space-time image illustrates the timing and location of the wave crests (see the dark gray ridges in Figure 2E). By examining trajectories of GFP-labeled cells (colored lines), we observe that the tracked cells travel with the high-density crests and reverse when and where two crests collide. Statistical analysis of the position and timing of cell reversals (dots) show that 75.0% (±2.6%) of all tracked reversals occur during wave crests collisions, matching ABM prediction (Figure 2F). Interestingly, some cells move through a counter-propagating wave crest without reversing and subsequently reverse with the next crest. This “wave-hopping” pattern explains the small peak at ∼12 min (twice the average reversal time) in Figure 2D and the more pronounced second peak in the distribution of the average distance travelled per reversal (Figure S8 E).

### Potential benefits of predatory rippling

#### A. Rippling facilitates cell expansion into prey areas

The benefits of rippling motility to *M. xanthus* cells during predation are unknown. However, the experimental results from Berleman et al. [13] indicate that rippling behavior correlates with an increase of the *M. xanthus* colony expansion rate over prey.

**Figure 3:**
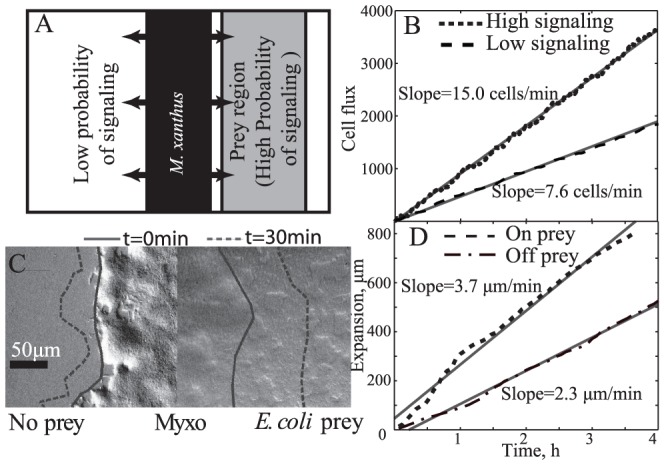
Ripples cause faster expansion of cells into the prey region. (A) Initial configuration of the ABM simulation with *M. xanthus* agents placed in the center area and thereafter expanded in both directions. On the right, a grey region represents the prey area where the probability of agents signaling to one another is increased (from *p_0_* = 0.03 to *p_0_* = 0.10) and therefore ripples are formed. (B) Using cell flux to measure the expansion [32], [33], we observed higher cell flux on prey (high signaling probability area) corresponding to a higher expansion rate on prey as demonstrated by the increased slope of regression line (grey). (C) Using ImageJ software to track the edge of a *M. xanthus* colony, the rate of the edge movement was computed. The solid line represents the edge of *M. xanthus* colony in this image and the dash line indicates its location 30 min later. (D) The experimentally observed expansion is plotted over time to show that the expansion rate over prey is about 1.6-fold larger than off the prey, as demonstrated by the increased slope of regression line (grey).

To test the effect of rippling motility on the expansion rate, we conducted a simulation in which agents, aligned along their X-axis, were placed into a central area from which they could expand in either direction (Figure 3A). To the right was a “prey region” in which the agents signaled during side-to-side contact with a probability large enough to form ripples. To the left, was an area containing no simulated prey, so the agents signaled one another during side-to-side contacts with a probability less than the threshold to induce rippling motility. As a result, ripples only formed in the “prey region”. For computational efficiency, the simulated colony expansion rate was evaluated based on the method of Wu et al. [32], [33], who concluded that cell flux is linearly related to the steady-state expansion rate. Therefore, we chose to compare cell flux in both directions to assess the effect of rippling motility on the expansion rate. The simulation results (Figure 3B) reveal a linear increase in the number of cells with time; the slope of this curve is a flux that predicts the cell-expansion rate. The result of the least-square linear fit of these data show a 2.0-fold increase in the slope in the rippling region. Therefore, ripple-inducing interactions increase the expansion rate by a factor of ∼2.0.

We tested this prediction experimentally by observing *M. xanthus* colony expansion on CTT nutrient agar with and without prey (Figure 3C and 3D, Video S5). The results indicate a 1.6-fold increase in the expansion rate over prey (see text S1 for analysis details). The most likely explanation for the difference between the predicted and experimental values for the colony expansion rate is that not all the *M. xanthus* cells in contact with the prey participate in rippling behavior. This is similar to the reason suggested for the observation that the ABM-simulated ripples are more pronounced compared to the experimental ripples (Figure 1 top vs. bottom).

The rippling-dependent increase in the expansion rate is understandable in terms of the basic model ingredients. As the predatory cells expand over the prey from one side, there is a gradient of *M. xanthus* cell density (at least near the leading edge). As a result, any cell is less likely to encounter a reversal-inducing side-to-side contact as it travels toward the prey and is more likely to encounter it as it moves away from the prey, which would cause the cell to reverse and travel toward the prey again. This increases the bias in the cell motility and as a result the cells spread over the prey-containing region faster. This behavior has clear physiological advantages in predation, as the cells are able to relatively quickly spread over prey before their potential competitors.

#### B. Rippling motility retains cells in the prey area longer

**Figure 4:**
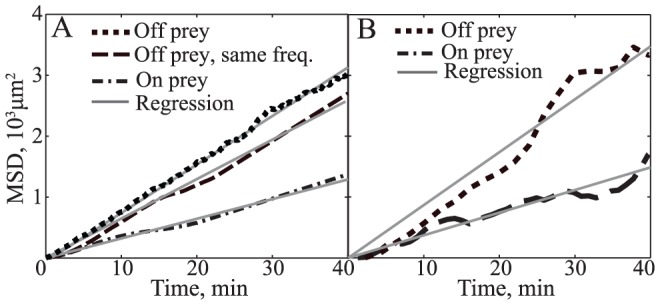
Comparison of mean square displacement (MSD) of *M. xanthus* cells on and off prey in both (A) ABM simulations and (B) experimental fluorescence microscope images. (A) MSD of simulated agents on prey (dotted-dash line) is linear with a slope of 32.2 µm^2^/min; for agents off prey (low signaling probability, *p_0_* = 0.03) with the same average reversal period the slope is 63.2 µm^2^/min; and for the cells off prey with the same reversal period and velocity as cells off prey in experiments (dotted line) the slope is 79.7 µm^2^/min. (B) For the experiments, ∼100 cells were tracked both on and off prey and Eq. (3) was used to calculate the MSD by averaging over all cells. The experimental MSDs of cells on prey increase linearly with time (dashed line) and can be fitted with a straight line with a slope of 38.5 µm^2^/min. Off prey (dotted line) is fitted with a straight line with a slope of 85.5 µm^2^/min. The gray solid lines in both panels are no-intercept linear regression fits of the MSDs.

Are there physiological benefits to rippling motility once *M. xanthus* cells completely and uniformly cover their prey? The ABM simulation data suggest that another potential benefit to rippling behavior is that the *M. xanthus* predator cells exhibiting rippling motility will remain in the prey regions longer. This is most likely due to the behavioral decrease in the randomness of cell motility resulting from the increase in the cell alignment and the periodicity of cell reversals. To examine this prediction, we computed a mean square displacement (MSD) of individual rippling and non-rippling agents (angled brackets denote averaging):
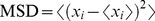
(3)To ensure a controlled comparison between rippling and non-rippling agents, we used identical values for the average speeds, reversal periods, and all parameters regarding rippling motility noise. As expected, the MSD increased linearly with time due to random diffusion-like drift with no bias (Figure 4). The slope, or effective diffusion coefficient, is smaller for agents that are rippling (Figure 4A, dotted-dash line) than those that are not rippling (Figure 4A, dashed line). The effective diffusion coefficient for non-rippling agents is about 2.0-fold greater than for the rippling agents. When controlling for the spontaneous rather than the average reversal period, the drift is increased to about 2.5 fold that of the rippling agents (Figure 4A, dotted line).

To test this prediction experimentally and to minimize any differences in behavior, we tracked rippling and non-rippling GFP-labeled cells within the same colony in the regions that were placed on top of prey cells or not, respectively. Eq. (3) was used to compute MSD for each representative cell. The observed results, depicted in Figure 4B, are quantitatively similar to those predicted by the ABM (Figure 4B). The non-rippling cells had a larger drift (2.2 fold) than the rippling cells.

A decreased MSD is anticipated for cells exhibiting rippling motility, because their synchronized cell movement and the resulting collective motility should be less noisy than individual cell motility. The rippling *M. xanthus* cells spend most of their time traveling back and forth within wave crests. They occasionally appear to skip a reversal, which allows them to join the next crest. Such synchronization also provides a physiological advantage, as a decrease in random drift makes it less likely that cells will move away from the prey region accidentally.

## Discussion

### Mechanistic basis of *M. xanthus* rippling behavior

*Myxococcus xanthus* cells self-organize into periodic bands of traveling waves, termed ripples, during multicellular fruiting body development and predation on other bacteria. Here we have used an approach that combines mathematical modeling with experimental observations to investigate the mechanistic basis of rippling behavior and its physiological role during predation. The resulting new mathematical model, which is more robust than previous models, is based on the recent observation of Mauriello et al. [8], that when counter-moving cells come into side-to-side contact, clusters of chemotaxis-like FrzCD receptors within the cells transiently align and thereafter one of the cells reverses. Our model shows that this side-to-side contact-mediated signaling is sufficient to induce rippling self-organization in a locally aligned cell population, assuming that there is a minimal refractory period during which the cells can not reverse again regardless of their signaling state. The existence of the refractory period has also been assumed in our previous model [19], [20] and this assumption is plausible as reversals are anticipated to require a significant reorganization of the cell-motility machinery [7], [34]. The existence of a refractory period also naturally follows from the dynamic properties of a negative-feedback oscillator (Frzilator), which was previously hypothesized to regulate cell reversals [35]. Altogether our modeling results suggest that the self-organization of cells into ripples during predation can be explained by the increased efficiency or higher probability of side-to-side signaling induced by the presence of prey macromolecules. This prediction is not tested directly in our experiments, but the emergent properties of simulated waves quantitatively match those in our predation experimental approach.

Our model builds on the detailed characterization of *M. xanthus* predatory rippling behavior by Berleman et al. [15], which showed that rippling motility occurs during predation on the variety of microorganisms and is induced by the presence of macromolecular substances. However, our model differs from the concept promoted by Berleman et al. [15] that ripples originate solely as an interaction of individual cells with macromolecules without any self-organizing intercellular interactions. In contrast, we propose that ripples result from the self-organization of cells into traveling wave patterns, which result from the intercellular signaling that is stimulated or facilitated by the presence of macromolecules. Indeed, in our experimental approach the macromolecules are likely to be distributed uniformly and their concentration is expected to vary very little during the typical wave period (∼10 min). Moreover, even if macromolecules induce the periodicity of *M. xanthus* cell motility as suggested by Berleman et al. [15], this would not be sufficient to induce ripples because their formation requires temporal and spatial synchronization of cellular behavior that is unattainable without cell-to-cell signaling.

Based on the previous modeling of *M. xanthus* developmental rippling behavior, one is prompted to ask: does the same mechanism control predatory and developmental rippling motility? Certainly this new model is similar to the previous mathematical models of developmental rippling, as they each consider that self-organization occurs when counter-moving cells interact to induce reversals [19], [20]. As expected, the new model is in good general agreement with the experimental patterns that were previously observed for developmental rippling motility [16], [18]. However, our tests reveal that this new side-to-side contact-mediated signaling model is much more robust, in that it can withstand realistic levels of variability in cell speed and reversal times (Figure S5 left panels). Specifically, when the level of randomness in cell motility consistent with the single-cell tracking experiments (fluctuations of velocity and reversal period over 30% of the mean value) is used in the pole-to-pole collision-mediated signaling model, the cells do not form ripples (Figure S5, bottom right panel). It is noteworthy that pole-to-pole signaling can result in more robust waves, if the cells are able to accumulate signals from multiple collisions and if signaling during the refractory period leads to a reduced reversal rate as the Frzilator model predicts [19]. However, for the new side-to-side contact-mediated signaling model, realistic rippling can be observed assuming only that single successful signaling events result in cellular reversals.

Furthermore, the experiments of Berleman et al. [13], [15] provided evidence indicating that developmental rippling occurs as a side effect of cell lysis during aggregation, which suggests that rippling motility is likely to be a response to the released macromolecules. Thus, we propose that our new side-to-side contact-mediated signaling model of rippling describes both predatory and developmental rippling. The new model therefore explains ripples without requiring the pole-to-pole exchange of the starvation-induced C-signal. This may be biologically justified for a number of reasons. First, to date no C-signaling receptor has been identified. Second, localization of CsgA to the cell poles has not been demonstrated directly. Third, the robustness of pole-to-pole signaling-mediated mechanism is questionable as the probability of this type of collision is low. However, as C-signaling mutants fail to display rippling motility [16], it would be interesting to investigate in future studies how C-signaling affects the FrzCD cluster alignment and whether C-signaling plays a role in predatory rippling.

### Quantitative and qualitative agreement between the new model and experimental observations

The main hypothesis of this new computational model is that rippling behavior is initiated by side-to-side contact-mediated signaling in the presence of prey cells. This hypothesis cannot be directly tested at this time, since we do not have a complete understanding of the specific biochemical mechanisms involved. However, we can rigorously test the model by comparing the model predictions with experimental data collected by us and others.

An important prediction of the model is that *M. xanthus* cells will reverse more frequently when prey is present. This agrees with our experimental observations (Table S3) and that of Berleman et al. [13]. Moreover, the resulting self-organization of cells into ripples provides various ways to quantitatively and qualitatively compare *in silico*-generated rippling motility with experimental observations.

A second prediction is that if the presence of prey stimulates this side-to-side contact-mediated signaling, then the rippling would only be observed in the regions where signaling is sufficiently probable, i.e. only in the regions covering prey. This is in good agreement with our observations (Video S6) and those of Berleman et al. [13], [15]. Indeed, our simulations show that the signaling probability can serve as a bifurcation parameter that induces a transition between the homogeneous cell distribution and the formation of ripples (Figure S6).

A third prediction is based on the timescale of rippling self-organization, which can be defined as the time it takes to generate ripples that consist of well-focused wave patterns, starting from an initially homogeneous cell population. Our model predicts that time to be of the order of 3 hrs, which is remarkably consistent with our experimental observations (Figure 1). The qualitative comparison of the time-lapse dynamics (Videos S1 vs. S2, S3 and S6) is also in good agreement. Interestingly, the time-scale of rippling origination in the experiments of Berleman et al. [13] is significantly longer (∼12 hrs). Although it is hard to pinpoint the source of this discrepancy, our model indicates that the cell density and the amount of noise in cell orientation can significantly affect the wave synchronization time.

A fourth prediction of the model is based on measuring the rippling wavelengths and correlating them to the parameters of individual cell motility. Our new model predicts a slightly modified relationship (Eq. (2) between wavelength, wave-crest width, individual cell speed, and reversal time as compared to the previously established [19], [20]. This new relationship is confirmed by our simulations and is in excellent agreement with the experimental measurements of wavelength (Figure 2 A and B, Table S2). The wavelength prediction is also compatible with previously reported measurements [13] and with the observations of Sliusarenko et al. [15], which show that cells moving in opposite directions tend to inter-penetrate one cell length before a reversal is triggered. Figure S7 shows the sequence of events that occur during two-crest collisions. This cartoon model indicates that once the cells at the front of each crest reverse, they signal to the cells following them, which results in a chain-reaction of signaling and reversal events. This cartoon also illustrates the importance of the refractory period, because once the cells at the front of the crest reverse, it is essential for them to keep signaling to other cells to reverse without reversing themselves.

A fifth prediction of the new model is based on tracking the cell reversals and locations of wave-crest collisions in time and space. Just as the model predicts (Figure 2F), the experimental results (Figure 2E) indicate that most reversals occur when and where two wave crests collide.

### The physiological role of rippling in predation

Our previous model of developmental rippling motility suggested [28] that periodic travelling waves can ensure a more regular distribution of fruiting-body aggregates at the colony edge, as seen in the submerged culture system of Welch et al. [18]. However, the physiological implications of this observation are unclear as the developmental aggregate distribution can be well organized even without rippling [36]. Furthermore, if rippling motility is predominantly a response to predation, what is its role in these situations? Berleman et al. [37] proposed two hypotheses. The first, termed the “grinder model” speculates that the movement of the waves of *M. xanthus* cells during rippling motility causes a physical disruption of the prey colony. The second, termed the “population control model” suggests that waves maximize the prey-predator contact area and push excess predator cells to the edges of the rippling area. Neither of these hypotheses is likely to be correct, based on the biophysics of this environment in which the very-low Reynolds number hydrodynamics will not allow temporary periodic perturbations to affect mixing or transport [38]. Nevertheless, our mathematical model suggests several alternatives for physiological benefits of rippling to predatory cells. These predictions are consistent with the experimental observations reported here and previously.

First, the model is in agreement with the observations of Berleman et al. [13] that during the expansion over prey, the presence of side-to-side contact-mediated signaling significantly facilitates the rate of *M. xanthus* cell spreading (Figure 3). As a result these cells cover their prey faster. This has obvious physiological benefits in the competitive soil environment. The observation is also consistent with our own experiments. Notably, this result does not require ripples per se, but only reversal-inducing signaling. However, our model indicates that side-to-side contact-mediated signaling is key for rippling self-organization and the other model ingredients can easily be justified by what is known about the biophysics of *M. xanthus* motility [7]. Furthermore, the increase in spreading also takes advantage of the cell-density gradient of *M. xanthus* cells that is generated by spreading at the leading edge. It is important to note that the rippling behavior does not require a density gradient of prey cells, as the alternative chemotaxis-based explanation would predict.

Second, the model predicts that cells that ripple in the absence of a cell-density gradient (i.e. when they are behind the leading edge of the swarm or once the prey is fully covered), would engage in less noisy and more periodic motion and as a result will have less of a random drift (Figure 4A). This effect would help the predatory cells to remain in the prey area for a longer time and to reduce random movement away from the prey. This prediction was confirmed by the cell-tracking assays (Figure 4B). Notably, this effect requires ripple formation, as the collective interaction of cells in the ripples leads to their synchronization. This effect is analogous to the well-known mathematical phenomena in which a collection of coupled noisy oscillators is less noisy than each oscillator on its own [39].

Third, it is likely that the formation of the ripples increases the cell alignment due to an increase in steric interactions in the denser crests. This prediction agrees with our observations and those of Berleman et al. [13]. However, it is worth noting that the causal relationship between rippling and alignment is not obvious, as ripples also require cell alignment. Therefore, it is likely that there is a positive self-reinforcing feedback loop between the formation of ripples and cell alignment: as cells align, ripples become more pronounced and their crests become more dense leading to further cell alignment. Although the physiological benefit of better alignment is not obvious, it may further enhance the rate of spreading, which contributes to the effects discussed above.

### Concluding remarks

Uncovering the mechanistic basis of spatial and temporal multicellular self-organization is a daunting task and a full understanding has not been achieved for even the best-studied model systems. Here, agent-based modeling, time-lapse fluorescence microscopy, and image quantification have been used synergistically to provide new insights into the mechanisms of *M. xanthus* self-organization into ripples. Our modeling demonstrates that a simple set of ingredients based on experimental observations is sufficient to produce rippling patterns. The subsequent experiments have tested a number of predictions based on the model and have allowed us to refine the model to achieve quantitative agreement with the experimental data. This type of combined approach is essential to further our understanding of self-organization in more complex systems such as development of multicellular organisms.

## Materials and Methods

### Agent-based modeling methods

ABM are widely used to computationally simulate emerging patterns formed by multiple agents. The ABM of *M. xanthus* rippling presented here is kept simple yet sufficiently flexible to accurately describe the experimentally observed behaviors of *M. xanthus* cells. The model is an extension of the earlier ABM [19] of *M. xanthus* self-organization that now incorporates a side-to-side contact-mediated signaling mechanism.

In this ABM, each agent represents a cell – a self-propelled rod on a 2-D surface with length of *L*, width of *w*, with a center position of (*x(t),y(t)*), and orientation 0≤*θ(t)*≤2π. Specifically, the agent length and width are constant throughout all simulations, whereas the center position and direction of movement are changed at each time step as the cells move and align. For each simulation, the time is updated by constant increments *δt*. The simulations are conducted on a fixed 2-D area in which all simulated moving agents are bounded. For most simulations periodic boundary conditions are imposed.

#### Cell movement

The agents' center positions are updated at each time step with both directed and random displacement
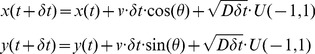
(4)Here *v* is the average cell speed, whereas *D* is the effective diffusion coefficient corresponding to speed fluctuations. These parameters are estimated from experimental data as discussed in Section 1.2. Here and below *U(a,b)* denotes a random number generated by a uniform distribution between *a* and *b*.

#### Cell reversals

To track the time between cell reversals, we introduced an internal timer phase variable, *φ(t)*, which has a range [*0,2π*). At each time step, the phase advances and when the phase increases past *π* and *2π*, the agents change their orientation *θ* by 180 degrees. In the absence of signaling, the reversal period is *T* and so the average phase speed is:
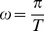
(5)As a result, without signaling, the phase at each time step is updated as follows:
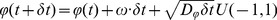
(6)where *D_φ_* is the effective diffusion coefficient of the phase, characterizing the fluctuation in phase velocity or equivalent fluctuations in reversal time. The value of term *D_φ_* is obtained by matching the reversal period distributions of the simulation and experimental observations (cf. Section 1.3). After *φ(t)* is computed at each time step, the following procedures are applied to periodically bind *φ(t)* within [*0,2π*) and ensure that the random fluctuations in phase near *π* and *2π* do not lead to additional reversals. The random fluctuations near *π* and *2π* are addressed first:
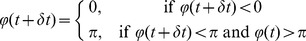
(7)Then the periodic boundary condition is applied:
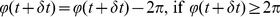
(8)When the phase increase exceeds *π* or 2*π*, the cells reverse direction by switching the polarities of the two ends:
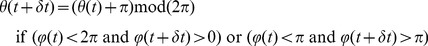
(9)


#### Side-to-side contact-mediated signaling mechanism and induced reversals

We propose that in addition to the signal-independent cell reversals, signal-induced early cell reversals are the key to ripple pattern formation. Recent experimental results show that during the side-to-side contact of two *M. xanthus* cells, their FrzCD clusters align and as a result one of the cells generally reverses [8]. Based on these experimental observations, we propose that side-to-side signaling is able to induce cell reversals. We developed an algorithm that incorporates the side-to-side contact-mediated signal mechanism into our ABM, including the spatial relationships between neighboring agents that allows for a side-to-side contact and the response if signaling occurs. For each selected agent, we first obtain a list of neighboring agents whose centers are inside a local square region centering around the selected agent center. Then, we apply the following procedures pairwise between the selected agent and one of its neighbors to determine if these two agents satisfy the conditions for side-to-side contact-mediated signaling and the response if the signaling occurs.The side-to-side contact-mediated signaling only occurs between two agents that are in contact, such that their long axes are aligned and they are traveling in opposite directions (see Figure S1) Therefore, we impose the following conditions to detect agents that make side-to-side contact:

1. To detect two agents with orientations *θ_1_* and *θ_2_* that have nearly parallel long axes, but travel in opposite directions, we identify cells for which:
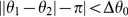
(10)For the simulations preformed, we chose threshold values of *Δθ_0_* to be 15 deg = 0.083*π*.
2. To determine if two nearly parallel agents are in contact and have significant overlap along their long axes, we include two additional cell-proximity requirements. For instance, if *(x_1_,y_1_)* denotes the center of the selected agent and *(x_2_, y_2_)* denotes one of its neighbors, then we define a vector 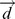 from the center of the selected agent to the neighbor 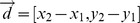. For two cells to make contact, we set limits on the projection of the vector 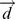 on the axis along the cell length and in the perpendicular direction. To define the average direction of two cells, we use unit vector 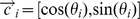 to represent the orientation of the *i*-th representative agents. As seen from Eq. (10), vectors 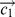 and 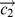 would point in nearly opposite directions, therefore the average orientation of two cells along their axis is determined by a vector
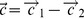
(11)The vector 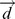 can then be projected into the average cell orientation defining the separation of cell centers along their average direction *d*_||_ and onto a perpendicular direction (distance *d_⊥_*) as follows (Figure S1):
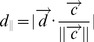
(12)

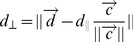
(13)where ∥*…*∥ is the vector norm and |…| is an absolute value. For two parallel cells to make side-to-side contact their *d_⊥_* must not exceed their cell widths:
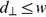
(14)At the same time, we propose that for efficient signaling at least 50% of the cells' long axes must overlap resulting in
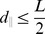
(15)

Conditions (10), (14) and (15) are calculated at every time step to determine all cell pairs that are in side-to-side contact and therefore capable of signaling. However, we assume that not every side-to-side contact will result in a signaling event, and therefore introduce a parameter, *p_0_*, which is the probability of signaling given the side-to-side contact. We assume the signaling is asymmetric and that the events of cell #1 signaling to cell #2 and vice versa are statistically independent. We also assume that *p_0_*<<1. These assumptions are motivated by the observations of Mauriello, et al. [8], that generally only one of the two cells reverses as a result of side-to-side contact. Therefore, for each cell in a side-by-side contact pair we generate a random number *U(0,1)* and only consider signaling to occur if *U(0,1)*<*p_0_*.

Every successful signaling event results in a reversal unless the cell is in a refractory period, i.e. has recently reversed. As each reversal event is associated with a change of cell polarity and requires reorganization of the cellular motors, it is natural to assume that there is a minimal reversal period during which a cell is unable to reverse again. This is termed the refractory period and is calculated using a phase-variable clock. After each reversal there is a sector *φ_0_* in the phase clock corresponding to an average refractory time *T_0_*, and *φ_0_ = ωT_0_*. During this time, an agent does not respond to the side-to-side signal, but it can always signal to other agents. In contrast, the agent is not refractory, if:
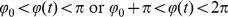
(16)then the agent is responsive to signals and will reverse. After the reversal, the phase variable is reset as follows:
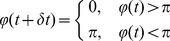
(17)As the signal can induce agent reversal, the orientation of the agent is reset as follows:
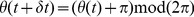
(18)


#### Cell alignment

Local cell alignment is essential for rippling. In this model, we chose to model cells as inflexible rods that align according to the equations of Sliusarenko et al [19]. More sophisticated alignment algorithms are not feasible here, because the behavior of up to 300,000 cells must be simulated. The alignment is modeled as
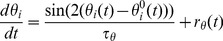
(19)where *τ_θ_* is the angle correlation time and *r_θ_(t)* is the random noise. 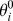 is the average nematic orientation of the cell's neighbors computed as follows. First, we define a neighboring region around each agent. To ensure fast computational speed, we use a square region with dimensions centered in the center of the selected agent. At each time step, for each agent *i*, we identify the list of *n_i_* neighbors with centers inside the square region. Second, we compute 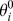 the average nematic orientation of neighbors as follows:
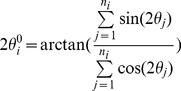
(20)Third, we discretize Eq. (19) using an implicit finite difference scheme to be solved iteratively
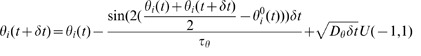
(21)If no reversal occurs, the computed *θ_i_(t+δt)* is the orientation of agent *i* at the next time step. Otherwise, we use equation (9) and (18) to further update the orientation.

#### Modeling parameter estimation

The parameters for the ABM simulations are summarized in Table S1. Whenever possible the parameters used were estimated directly or indirectly from the experimental data obtained in our conditions. For example, the analysis of individual cell movement described above provides both average cell characteristics (such as average velocity and reversal period) and their population distributions. The agent velocity *v* used in this ABM simulation is the average velocity calculated in the above analysis. The diffusion coefficient *D*, which characterizes the random fluctuation in agent movement, is chosen such that the variance of the instant velocity distribution of the ABM simulation matches the results of the experimental data analysis. Note that the experimentally observed random fluctuations along the *x* direction and *y* direction are almost identical. As a result, only one value *D* is used to represent the noise level in cell movement. In our ABM simulations of rippling, varying the refractory period changes the average reversal period. Thus, the refractory period was chosen to fit the average reversal period in the ABM to that in the experimental observations of rippling cells. The average reversal period of non-rippling cells observed experimentally was chosen as the natural reversal period *T* in our ABM and the phase speed *ω* was calculated using equation (5). The diffusion coefficients in the reversal period *D_φ_* were chosen by matching the distribution of reversal periods of the ABM simulations to the experimentally observed distribution. The phase variable *φ_0_* in the ABM simulations was chosen so that 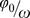 would equal the selected refractory period. There are also parameters that cannot be directly estimated experimentally, but can be defined based on the simulation results. For example, the random noise level *D_θ_* is assigned such that the initially aligned population of cells remains aligned.

### Experimental methods

#### Cell growth and development

For all experiments *M. xanthus* strains DK1622 (wild-type strain) and Mx477 (DK1622 P*_pilA_*:GFP) were grown overnight in CTT broth (1% Difco Casitone, 10 mM Tris-HCl pH 8.0, 8 mM MgSO_4_ and 1 mM KHPO_4_ pH 7.6) at 32°C with shaking. When *M. xanthus* cells reached mid-log phase (4×10^8^ cells/ml, 100 Klett units), they were centrifuged at 6,000× g and resuspended to Klett 250 in TPM buffer (CTT without Casitone). The two strains were mixed to achieve a 5∶1000 cell ratio of Mx477 to DK1622, respectively. To control the population of prey for the experiment, we used the thymine auxotrophic *E. coli* strain AB2497 (AB1157 *thyA12 deoB6*). Prey growth was described in more detail by Fonville et al. [40]. Briefly, *E. coli* cells were grown at 37°C in M9 medium with 50 mg/ml thymine, 0.1% glucose and 0.5% casamino acids. Prior to experiments the cells were diluted to an OD_600_ of 0.1 in M9 medium+glucose (no thymine) and allowed to grow for 1 hr. For fluorescence imaging the prey cells were treated with 1 ug/mL of DAPI for 20 min.

#### Microscopic imaging

For microscopy *M. xanthus* and prey cells were placed on ½ CTT (CTT broth with 0.5% Casitone) 1.5% agar in a 10 cm petri dish. A 7-uL drop of each culture was placed on ½ CTT agar, so that the edges of the colonies would be less than 1 cm apart, but did not touch. Cells were allowed to acclimate for at least 2 hrs prior to imaging. The agar dish with cells was inverted onto a microscope slide for imaging. Cells were imaged with an Olympus 81X inverted fluorescence microscope with a Hamamatsu HD camera. Moist Kimwipes were used to maintain humidity and reduce evaporation and cells were maintained at 28–30°C using a custom-built Precision Weather Station.

To obtain information on individual live cells, we obtained time-lapse images of fluorescently-labeled *M. xanthus* in a mixed population of cells (99.5% wild type DK1622 and 0.5% Mx477 P*_pil_*-GFP). Images of a mixed population of cells on nutrient agar were collected every 1 min for up to 4 hrs. ImageJ software [41] and custom Matlab code was used to track the *x*, *y* coordinates of individual cells in a given frame number n: *x(n)* and *y(n)*. The motility parameters of cells were calculated from these data (see Figure 2 C and D, Figure S8 D–G). The details of image analysis and quantification procedures are described in Text S1.

## Supporting Information

Figure S1 **Side-to-side contact signaling in the ABM simulations.** The side-to-side contact in the ABM simulations is defined by three parameters: 1) the perpendicular (to cell orientation) distance between the center of the two agents (*d*_⊥_); 2) the parallel distance between the center of the two agents (*d*_∥_); and 3) the angle formed by the two agents (*Δθ* in this figure). *L* represents the length of the cells and *v* represents velocity.
(PDF)

Figure S2 **The new side-to-side contact-mediated signaling mechanism is compared to the previous pole-to-pole collision-mediated signaling mechanism.** Although both mechanisms can produce ripples at a low noise level, the side-to-side contact-mediated signaling mechanism is significantly more robust. To produce ripples in the ABM, the head-to-head collision-mediated signaling mechanism must have 100% signal probability, whereas the side-to-side contact-mediated signaling mechanism only needs 10% signal probability. When the noise level is increased to match the value obtained in the experiments (standard deviation is about 25% of the mean), only the side-to-side contact-mediated signaling mechanism can produce ripples (bottom panels); the head-to-head collision signal does not produce visible ripples even with 100% signal probability.
(PDF)

Figure S3 **The distribution of reversal parameters in an agent population.** As a result of fluctuations in phase-clock speed, the agents in our simulation show stochastically variable refractory period (Panel A) and a native reversal period (Panel B). The mean and standard deviations are as indicated. Simulations for 30 cells were done as indicated in the Materials and Methods section but without signaling (signaling probability = 0) to correspond to isolated cells that cannot signal to one another.
(PDF)

Figure S4 **Variation of the ABM ingredients can affect wave formation.** (A) Waves are destroyed if cells signal to one-another irrespective of their gliding direction, *i.e.* cells going in the same and in the opposite direction signal with the same probability. (B) Waves are destroyed if only cells moving in the same direction signal to one-another. (C) Waves form when only oppositely moving cells signal to one-another – the same assumption as in the rest of the simulations. (D) Same as Panel C, but the signaling event is symmetric: when two cells signal to one-another they both reverse unless they are in a refractory period. As a result, waves are formed and appear very similar to those with asymmetric signaling used in the rest of the simulations. (E,F) Reduction of the refractory period impairs the wave patterns. (E) Waves disappear if the reversal period is reduced 10-fold from the value used in all the main text simulations (mean value of about 25 s). (F) Wave patterns become obscure if the reversal period is reduced 3-fold from the value used in all the main text simulations (mean value of about 1 min). All the panels are of the same scale: the simulation domain is 500 µm×100 µm, which is slightly reduced from the main text simulations for computational efficiency, the scale bar is 50 µm.
(PDF)

Figure S5
**Ripples are resistant to variations of the minimal overlap** 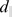 **threshold required for signaling, but become less focused with an increase of this threshold.** (See Eq.(15) and Methods section for definitions). Signaling only appears when 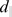 is below a given threshold of (A) 0.8*L*, (B) 0.7*L*, (C) 0.6*L*, (D) 0.5*L*, as in the rest of the simulations: (E) 0.4*L*, (F) 0.3*L*, and (G) 0.2*L*. The cell length is *L* = 7 µm. All the panels are of the same scale:, simulation domain is 500 µm×100 µm, which is slightly reduced from the main text simulations for computational efficiency; the scale bar is 50 µm.(PDF)

Figure S6 **Signal probability is a good bifurcation parameter to control self-organization into ripples.** (A–D) Wavelet transforms are a sensitive measure to detect ripples. (A) The wavelet coefficient from a wavelet transform of an experimental image that contains ripples. (B) The wavelet coefficient from a wavelet transform of an experimental image without ripples. (C) The wavelet coefficient from a wavelet transform of an image with ripples from the ABM simulation. (D) The wavelet coefficient from a wavelet transform of an image without ripples from the ABM simulation. (E) The order parameter (see Text S1) is computed from the wavelet coefficients as an indication of the presence of ripples. The order parameter is close to zero when there are no ripples and greater than 0.4 when ripples are present. The error bar is computed from 10 independent simulations. This figure shows that the signal probability serves as a bifurcation switch of the *M. xanthus* rippling pattern.
(PDF)

Figure S7 **Individual cells can form ripples as they reverse their direction during crest edge collisions produced from the ABM simulation data.** The directions of the arrows indicate the direction of cell movement. Pairs of cells engaged in side-to-side signaling are circled. Cells travelling to the right are red and cells travelling to the left are blue. (A) Two opposing waves approach each other and the cells begin to make side-to-side contacts. (B) The initial stage of the collision of the two wave crests. Three pairs of cells are engaged in signaling (circled). As a result of the signaling, some cells reverse and others continue without changing their direction. (C) Two more signaling events occur between reversed cells and their previous followers in the same crests. (D) The two waves have completed their collision and reversed their direction. Note that in some examples both signaling cells reverse their directions due to interactions with other cells (not shown).
(PDF)

Figure S8 **Results of experimental data analysis.** (A) The background image is acquired from a DIC microscopic image that shows a rippling pattern. Individual cell trajectories of 11 cells are shown in blue. The same set of images is the source of the background image and the cell trajectories. The cells appear to move predominately in one direction, which is the same as the wave direction. The red arrow shows the direction of wave movement, which is computed from the principle component analysis (PCA). (B) All the cell coordinates are centered by subtracting the average position of each cell. Then, the trajectories of all cells are placed together and the PCA is applied. The dash line is the regression line. (C) A schematic diagram showing two situations in which cells change directions in several consecutive frames. In one case, the cell changes direction eventually (one of the points is an actual reversal) and in the other, the cell continues in the same direction. (D) A trajectory of a typical cell traveling with the rippling wave crest. The red dots denote where cellular reversals occur. (E) The distribution of distances that cells travel between reversals. (F) A trajectory of a typical cell that is on prey, but does not travel with the wave crest (non-rippling cell). (G) A trajectory of a typical cell that is not on prey.
(PDF)

Table S1 **Parameters used in the simulations.**
(PDF)

Table S2 **Experimental data for individual cell motility parameters and the resulting wavelengths is consistent with the** **Eq. (2)** **.**
(PDF)

Table S3 **Motility parameters for cells tracked on and off prey.**
(PDF)

Text S1 **Quantification and images analysis of microscopy and of ABM simulation data.**
(PDF)

Video S1 **ABM simulation shows the time-lapse dynamics of the formation of ripples starting from initially homogeneous distribution.** The scale bar is 100 µm. The movie is composed of ∼900 simulation snap-shots taken every 20 seconds over the period of 5 hours. The resulting movie is compiled at 20 fps.
(MP4)

Video S2 **Experimental time-lapse movie of** ***M. xanthus*** **ripples from superimposed DIC (gray-scale background) and fluorescence microscopy (false-colored in green) images.** About 0.5% of the cells are fluorescently labeled to allow easy tracking. The movie is composed of ∼140 frames taken every minute over the period of ∼2.5 h. The resulting movie compiled at 10 fps.
(AVI)

Video S3 **Experimental time-lapse DIC channel movie showing the dynamics of ripple initiation following the complete coverage of the prey.** The movie starts from the time when *M. xanthus* fully cover the prey in the field of view. The movie is composed of ∼150 frames taken every two minutes over the period of ∼5 h. The resulting movie is compiled at 5 fps.
(AVI)

Video S4 **Same as Video S1 but with parameters corresponding to Berleman et al. (2008).** The movie is composed of ∼850 simulation snap-shots taken every 20 s over the period of ∼4.5 h. The resulting movie is compiled at 20 fps.
(MP4)

Video S5 **Side-by-side comparison of** ***M. xanthus*** **colony expansion on and off prey.** The black lines label the extending colony boundary whereas stationary grey lines represent the boundary at the initiation of the movie. Each movie is composed of 31 frames taken every 4 min over the period of ∼2 hours. The resulting movie is compiled at 5 fps.
(AVI)

Video S6 **Experimental line indicating that rippling only occurs in direct contact with prey under our conditions.** The red line approximately marks the edge of the region where prey was initially placed. The scale bar is 50 µm. The movie is composed of 21 frames taken every two minutes over the period of ∼40 min. The resulting movie is compiled at 2 fps.
(AVI)
