# Supplementary figures and images for: The Mechanistic Basis of Myxococcus xanthus Rippling Behavior and Its Physiological Role during Predation
Source: PLoS Comput Biol. 2012 Sep 27;8(9):e1002715. doi: 10.1371/journal.pcbi.1002715 (PMC3459850; doi:10.1371/journal.pcbi.1002715)

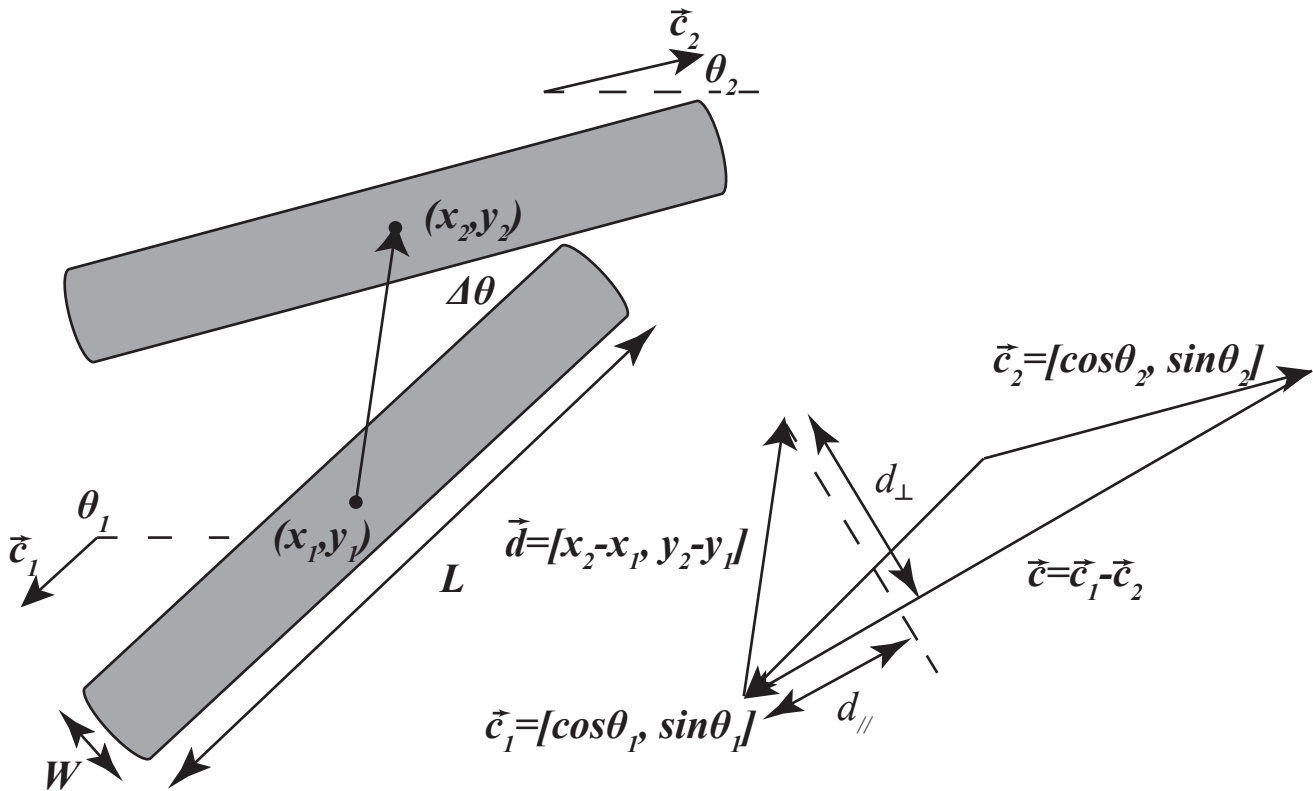

Supplement: Figure S1 — Side-to-side contact signaling in the ABM simulations. The side-to-side contact in the ABM simulations is defined by three parameters: 1) the perpendicular (to cell orientation) distance between the center of the two agents (d ⊥); 2) the parallel distance between the center of the two agents (d ∥); and 3) the angle formed by the two agents (Δθ in this figure). L represents the length of the cells and v represents velocity. (PDF) [file pcbi.1002715.s001.pdf]

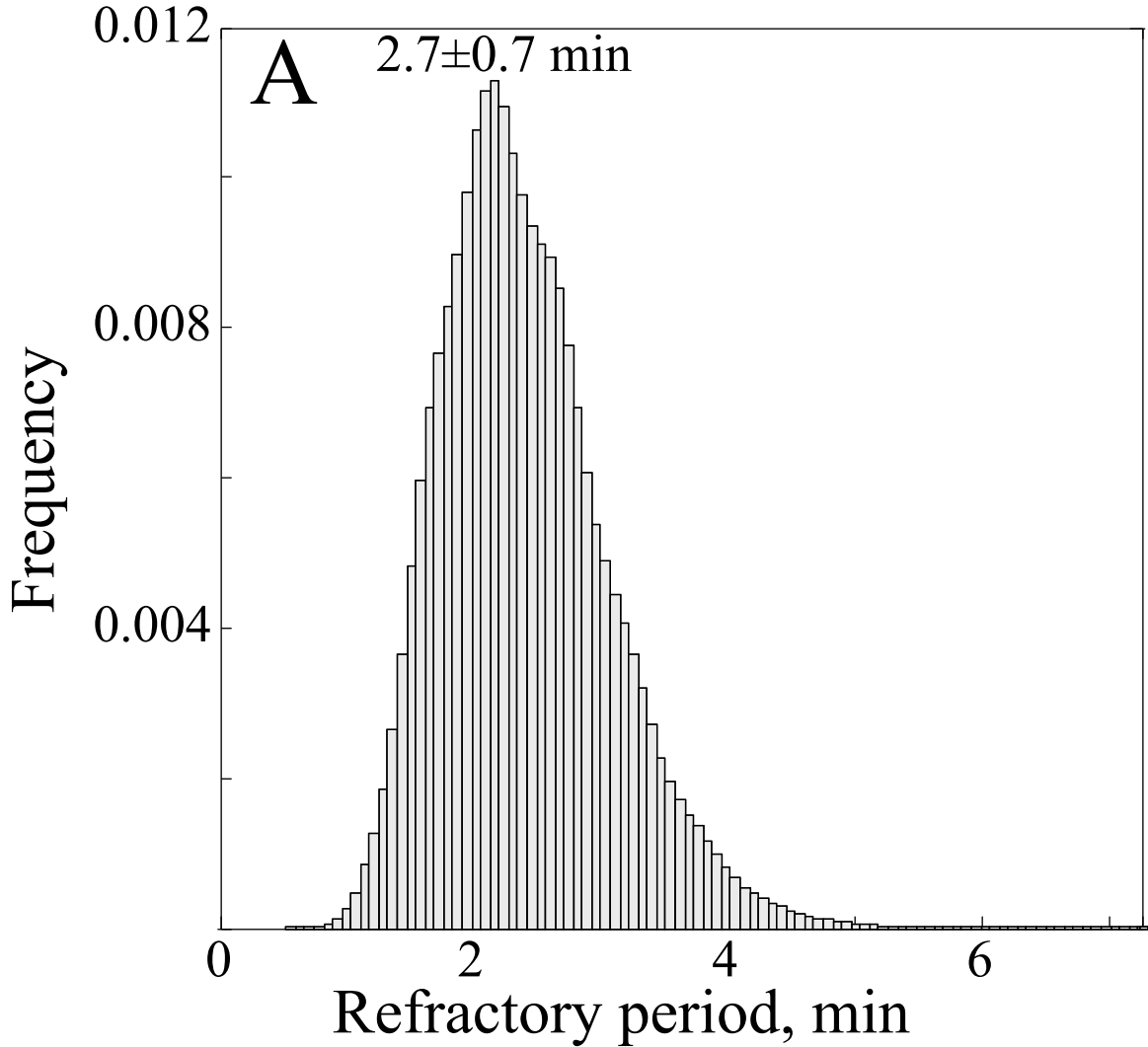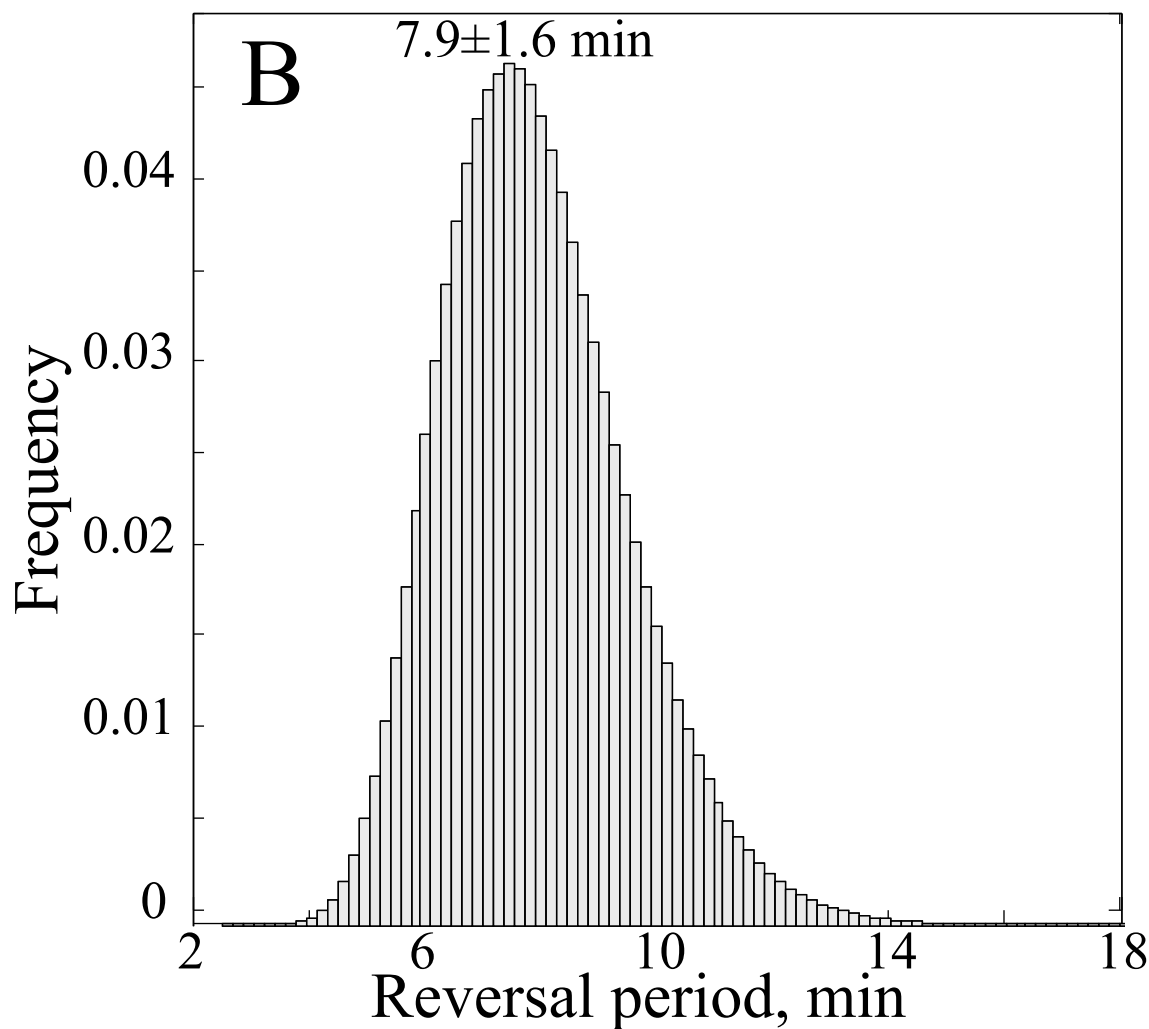

Supplement: Figure S3 — The distribution of reversal parameters in an agent population. As a result of fluctuations in phase-clock speed, the agents in our simulation show stochastically variable refractory period (Panel A) and a native reversal period (Panel B). The mean and standard deviations are as indicated. Simulations for 30 cells were done as indicated in the Materials and Methods section but without signaling (signaling probability = 0) to correspond to isolated cells that cannot signal to one another. (PDF) [file pcbi.1002715.s003.pdf]

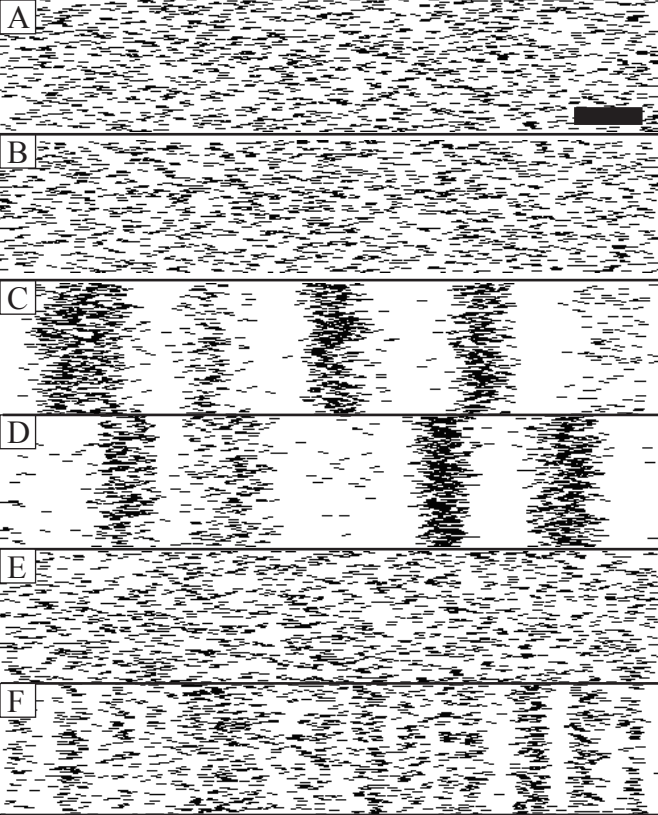

Supplement: Figure S4 — Variation of the ABM ingredients can affect wave formation. (A) Waves are destroyed if cells signal to one-another irrespective of their gliding direction, i.e. cells going in the same and in the opposite direction signal with the same probability. (B) Waves are destroyed if only cells moving in the same direction signal to one-another. (C) Waves form when only oppositely moving cells signal to one-another – the same assumption as in the rest of the simulations. (D) Same as Panel C, but the signaling event is symmetric: when two cells signal to one-another they both reverse unless they are in a refractory period. As a result, waves are formed and appear very similar to those with asymmetric signaling used in the rest of the simulations. (E,F) Reduction of the refractory period impairs the wave patterns. (E) Waves disappear if the reversal period is reduced 10-fold from the value used in all the main text simulations (mean value of about 25 s). (F) Wave patterns become obscure if the reversal period is reduced 3-fold from the value used in all the main text simulations (mean value of about 1 min). All the panels are of the same scale: the simulation domain is 500 µm×100 µm, which is slightly reduced from the main text simulations for computational efficiency, the scale bar is 50 µm. (PDF) [file pcbi.1002715.s004.pdf]

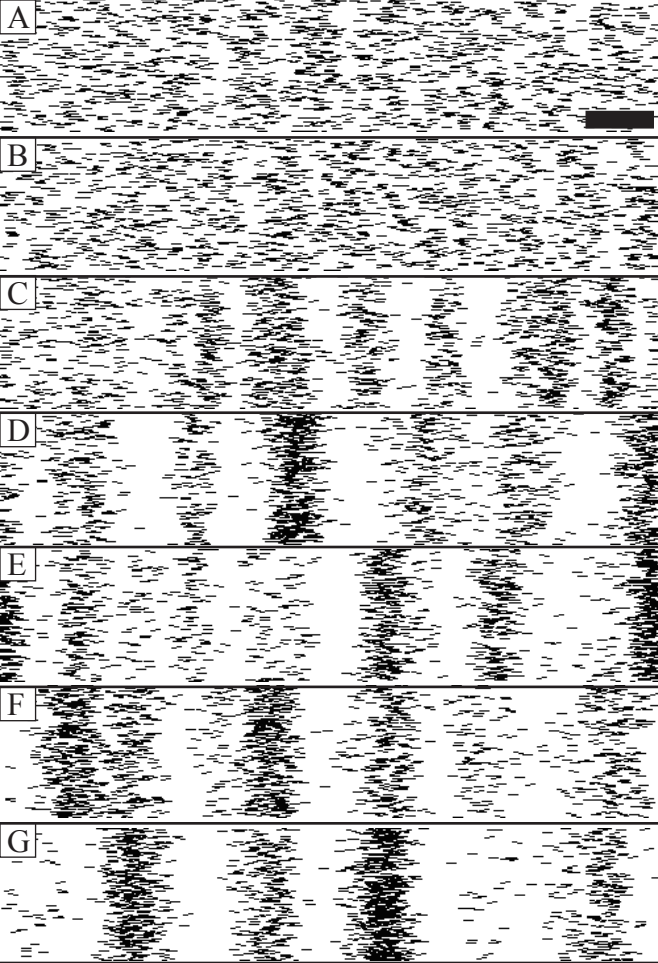

Supplement: Figure S5 — Ripples are resistant to variations of the minimal overlap threshold required for signaling, but become less focused with an increase of this threshold. (See Eq.(15) and Methods section for definitions). Signaling only appears when is below a given threshold of (A) 0.8L, (B) 0.7L, (C) 0.6L, (D) 0.5L, as in the rest of the simulations: (E) 0.4L, (F) 0.3L, and (G) 0.2L. The cell length is L = 7 µm. All the panels are of the same scale:, simulation domain is 500 µm×100 µm, which is slightly reduced from the main text simulations for computational efficiency; the scale bar is 50 µm. (PDF) [file pcbi.1002715.s005.pdf]

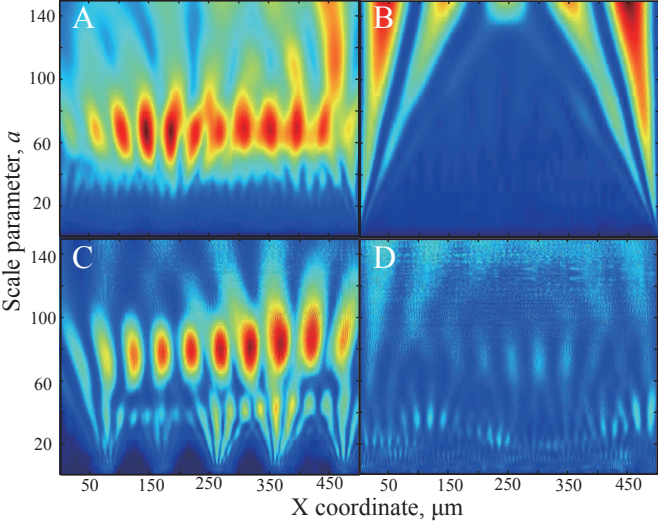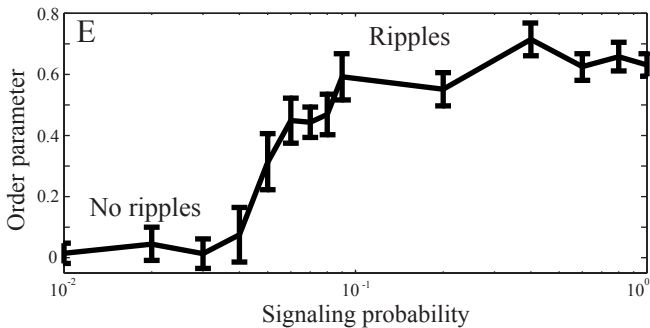

Supplement: Figure S6 — Signal probability is a good bifurcation parameter to control self-organization into ripples. (A–D) Wavelet transforms are a sensitive measure to detect ripples. (A) The wavelet coefficient from a wavelet transform of an experimental image that contains ripples. (B) The wavelet coefficient from a wavelet transform of an experimental image without ripples. (C) The wavelet coefficient from a wavelet transform of an image with ripples from the ABM simulation. (D) The wavelet coefficient from a wavelet transform of an image without ripples from the ABM simulation. (E) The order parameter (see Text S1) is computed from the wavelet coefficients as an indication of the presence of ripples. The order parameter is close to zero when there are no ripples and greater than 0.4 when ripples are present. The error bar is computed from 10 independent simulations. This figure shows that the signal probability serves as a bifurcation switch of the M. xanthus rippling pattern. (PDF) [file pcbi.1002715.s006.pdf]

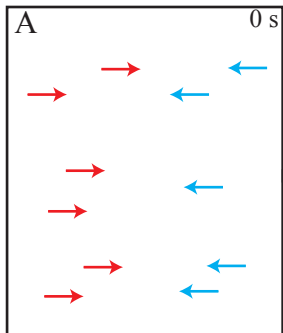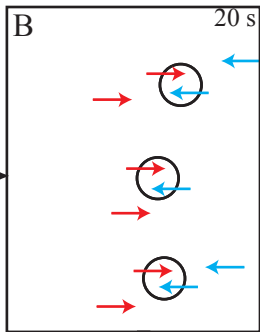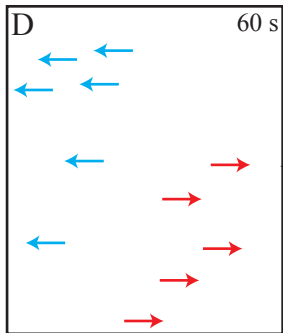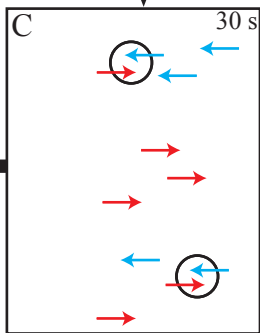

Supplement: Figure S7 — Individual cells can form ripples as they reverse their direction during crest edge collisions produced from the ABM simulation data. The directions of the arrows indicate the direction of cell movement. Pairs of cells engaged in side-to-side signaling are circled. Cells travelling to the right are red and cells travelling to the left are blue. (A) Two opposing waves approach each other and the cells begin to make side-to-side contacts. (B) The initial stage of the collision of the two wave crests. Three pairs of cells are engaged in signaling (circled). As a result of the signaling, some cells reverse and others continue without changing their direction. (C) Two more signaling events occur between reversed cells and their previous followers in the same crests. (D) The two waves have completed their collision and reversed their direction. Note that in some examples both signaling cells reverse their directions due to interactions with other cells (not shown). (PDF) [file pcbi.1002715.s007.pdf]

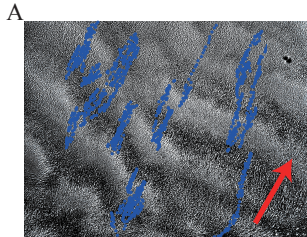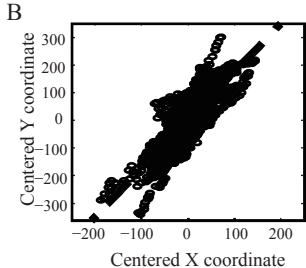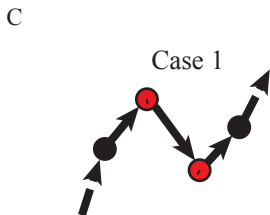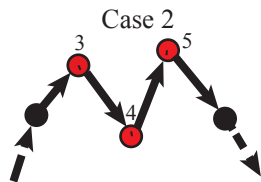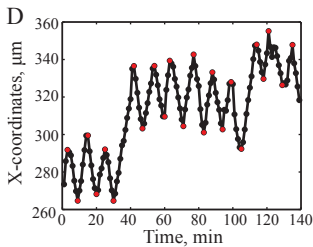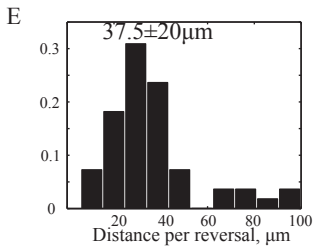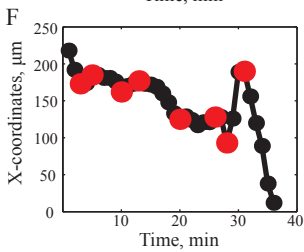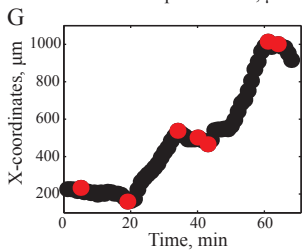

Supplement: Figure S8 — Results of experimental data analysis. (A) The background image is acquired from a DIC microscopic image that shows a rippling pattern. Individual cell trajectories of 11 cells are shown in blue. The same set of images is the source of the background image and the cell trajectories. The cells appear to move predominately in one direction, which is the same as the wave direction. The red arrow shows the direction of wave movement, which is computed from the principle component analysis (PCA). (B) All the cell coordinates are centered by subtracting the average position of each cell. Then, the trajectories of all cells are placed together and the PCA is applied. The dash line is the regression line. (C) A schematic diagram showing two situations in which cells change directions in several consecutive frames. In one case, the cell changes direction eventually (one of the points is an actual reversal) and in the other, the cell continues in the same direction. (D) A trajectory of a typical cell traveling with the rippling wave crest. The red dots denote where cellular reversals occur. (E) The distribution of distances that cells travel between reversals. (F) A trajectory of a typical cell that is on prey, but does not travel with the wave crest (non-rippling cell). (G) A trajectory of a typical cell that is not on prey. (PDF) [file pcbi.1002715.s008.pdf]
