# Supplementary material for: The Mechanistic Basis of Myxococcus xanthus Rippling Behavior and Its Physiological Role during Predation
Source: PLoS Comput Biol. 2012 Sep 27;8(9):e1002715. doi: 10.1371/journal.pcbi.1002715 (PMC3459850; doi:10.1371/journal.pcbi.1002715)
Supplement: Figure S2 — The new side-to-side contact-mediated signaling mechanism is compared to the previous pole-to-pole collision-mediated signaling mechanism. Although both mechanisms can produce ripples at a low noise level, the side-to-side contact-mediated signaling mechanism is significantly more robust. To produce ripples in the ABM, the head-to-head collision-mediated signaling mechanism must have 100% signal probability, whereas the side-to-side contact-mediated signaling mechanism only needs 10% signal probability. When the noise level is increased to match the value obtained in the experiments (standard deviation is about 25% of the mean), only the side-to-side contact-mediated signaling mechanism can produce ripples (bottom panels); the head-to-head collision signal does not produce visible ripples even with 100% signal probability. (PDF) [file pcbi.1002715.s002.pdf]

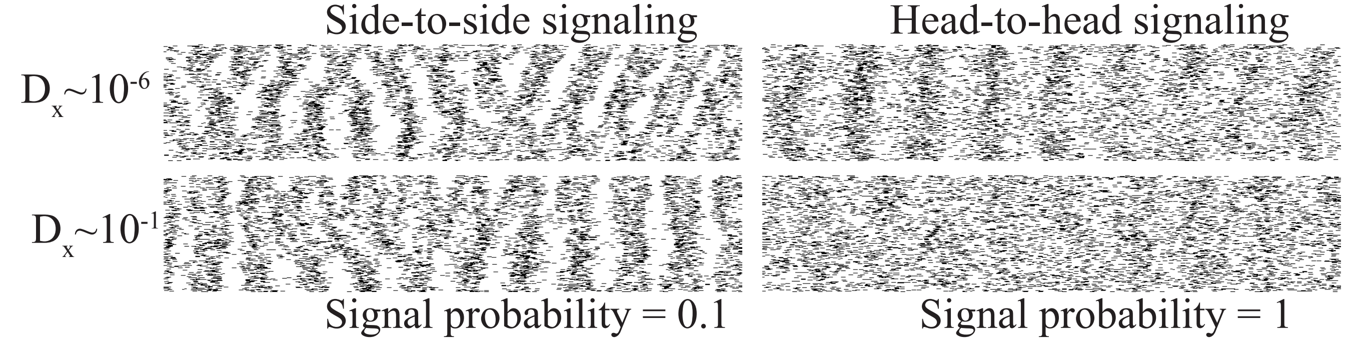
Side-to-side signaling
Head-to-head signaling
D_x~10^-6
D_x~10^-1
Signal probability = 0.1
Signal probability = 1
