# Supplementary material for: The Mechanistic Basis of Myxococcus xanthus Rippling Behavior and Its Physiological Role during Predation
Source: PLoS Comput Biol. 2012 Sep 27;8(9):e1002715. doi: 10.1371/journal.pcbi.1002715 (PMC3459850; doi:10.1371/journal.pcbi.1002715)
Supplement: Table S1 — Parameters used in the simulations. (PDF) [file pcbi.1002715.s009.pdf]

Table S1 Parameters used in simulations

Variable name	Description	Value	Source
ρ	Cell density	0.3 /μm^2	The cell density is corresponding to one layer of densely packed cells
δt	Simulation time-step	5 s	
w	Cell width	0.5 μm	From observation of individual cells
L	Cell length	7 μm	From observation of individual cells
v	Agent velocity	6 μm/min	Average cell velocity observed in experiments
D	Spatial diffusion coefficient	0.1 μm^2/s	Obtained by matching instant cell velocity distributions in ABM simulations and experimental data analysis
T	Natural reversal period of agents	8 min	Average cell reversal period observed in experiments of non-rippling cells
D_φ	Phase diffusion coefficient	10^{-4} rad^2/s	Obtained by matching reversal period distributions in ABM simulations and experimental data analysis
T_0	Refractory time	2.6 min	Obtained by matching the average reversal period in ABM simulations and experimental observations of ripping cells.
$\Delta\theta_0$	Angle difference threshold for contact detection	0.0833π	
τ_θ	Anglular correlation time	50 s	Adapted from the work of Sliusarenko et al.[19]
D_θ	Angle diffusion coefficient	10^{-4} rad^2/s	Adapted from the work of Sliusarenko et al. [19]
p_0	Signal probability per time-step (“on prey”)	0.1	Toned to match the experimental observed pattern
p_0	Signal probability per time-step (“off prey’)	0.03	Chosen below the ripple-inducing threshold (Figure S3)
