# Supplementary material for: The Mechanistic Basis of Myxococcus xanthus Rippling Behavior and Its Physiological Role during Predation
Source: PLoS Comput Biol. 2012 Sep 27;8(9):e1002715. doi: 10.1371/journal.pcbi.1002715 (PMC3459850; doi:10.1371/journal.pcbi.1002715)
Supplement: Table S2 — Experimental data for individual cell motility parameters and the resulting wavelengths is consistent with the Eq. (2) . (PDF) [file pcbi.1002715.s010.pdf]

Table S2: Experimental data on individual cell motility parameters and resulted wavelengths is consistent with the Eq.(2)

Data source	Cell speed	Reversal period	Wave-crest width	Predicted wavelength	Measured wavelength
Our measurements (main text figure)	6.0±1.5μm/min	6.6±2.4 min	10 ±5μm	99.2μm	82±15μm
Our measurements[1]	2.9±1.7 m/min	6.0±6.0 min	5±2μm	44.8μm	40±12μm
Our measurements[1]	3.0±1.7μm/min	4.5±4.3 min	2±3μm	31.0μm	28±4μm
Ref. [13] [2]	~3.0μm/min	~8.0 min	~10μm	68.0μm	~ 70μm

[1] We selected two movies displaying different wavelength for detailed analysis and tracking. Variations in cell speed and reversal period are due to differences in agar and prey density.
[2] Data on extracted from Figures and text of Ref. [13].
