# Supplementary material for: The Mechanistic Basis of Myxococcus xanthus Rippling Behavior and Its Physiological Role during Predation
Source: PLoS Comput Biol. 2012 Sep 27;8(9):e1002715. doi: 10.1371/journal.pcbi.1002715 (PMC3459850; doi:10.1371/journal.pcbi.1002715)
Supplement: Table S3 — Motility parameters for cells tracked on and off prey. (PDF) [file pcbi.1002715.s011.pdf]

Table S3: Motility parameters for cells tracked on and off prey

Experimental observations	Cell speed	Reversal period
On prey (rippling)	6.0±1.5 μm/min	6.6±2.4 min
Off prey(no rippling)	6.1±1.7 μm/min	8.4±7.5 min*

* Part of the variability is associated with technical difficulties in automatic reversal detection in non-aligned population.
