# Supplementary material for: The Mechanistic Basis of Myxococcus xanthus Rippling Behavior and Its Physiological Role during Predation
Source: PLoS Comput Biol. 2012 Sep 27;8(9):e1002715. doi: 10.1371/journal.pcbi.1002715 (PMC3459850; doi:10.1371/journal.pcbi.1002715)
Supplement: Text S1 — Quantification and images analysis of microscopy and of ABM simulation data. (PDF) [file pcbi.1002715.s012.pdf]

Text S1: Quantification and images analysis of microscopy and of ABM simulation data.

Tracking cells and measuring motility parameters

<u>Instant cell displacements and speeds:</u> The instant displacement of each cell between two consecutive frames was computed as:

$$d(n) = \sqrt{(x(n) - x(n-1))^2 + (y(n) - y(n-1))^2} \tag{S.1}$$

The instant cell speed was computed as

$$v(n) = \frac{d(n)}{\delta t} \tag{S.2}$$

where δt is the time interval between successive frames.

<u>Removal of temporarily immotile cells:</u> Analysis of the cell trajectories revealed several instances in which the fluorescent cells stop moving for extended periods of time (> 5 min). These data were removed from the trajectories. This was accomplished by identifying those cells for which the instant cell speed was < 0.2 µm/min (less than 5% of the average speed) for continuous time intervals of 5 min or longer and excluding these intervals from the motility statistics.

<u>Detection of cell reversals:</u> To calculate the statistics of the cell reversal periods we needed an algorithm that robustly detected cellular reversals, but ignored changes in cell velocity associated with random motion and/or turning. For our experimental approach the problem was simplified by the observation that cells are predominantly aligned and therefore travel in the same direction (see Figure S5A). This direction is generally not parallel to the image boundaries. Therefore the images were rotated to ensure that the majority of the cells move along their X-axis.

To determine the direction in which the cells are predominantly aligned, we applied principal component analysis (PCA) to the cell trajectories obtained from the individual cell tracking data. PCA was used in multivariate analysis to determine the important elements within the data set. The largest variance is the first principal component. Here, we rotated the first principal component to make it parallel to the X-axis. As a result, most cell move parallel to the X-axis.

After rotation, the reversal points in the cell trajectories can be identified based on the sign changing points in the time-derivative of the coordinates along the first principal component ($\hat{x}_i(n)$). The sign of the X-axis projection of the cell velocity is:

$$s(n) = \text{sign}(\hat{x}_i(n) - \hat{x}_i(n-1)) \quad \text{(S.3)}$$

When s=+1 a given cell travels along the principal axis in the positive direction, whereas when s =−1 it travels in the opposite direction. The points where the cell velocity changes its sign therefore can be calculated as the non-zero elements of a sign-changing vector *sc:*

$$sc(n) = s(n) - s(n-1) \quad \text{(S.4)}$$

However, not all of the sign changing points are the real reversal points, as cellular motility noise and tracking errors can result in false-positives. Therefore, additional analysis is applied to the sign changing data *sc* to find the real reversal points.

By examining *sc,* it became clear that sometimes there are several sign changing points that occur consecutively during a very short period of time. Cell behavior during these periods is termed "tumbling". The algorithm below was used to address these tumbling events. For example, Figure S5C shows an application of the algorithm

i. The range of indices with successive non-zero values in *sc* was determined. These are the tumbling events (Figure S5 C). The red dots indicate a tumbling event.
ii. The number of sign changing points for each tumbling event was determined. For example, in Figure S5 C, there are two sign changing points in Case 1 and three in Case 2.
iii. The tumbling events are separated into two cases. In Case 1, the cell continues in the same direction after the tumble as before the tumble. This is reflected in an even number of sign-changing points for the tumble (see Figure S5 C left panel). In Case 2, the cell changes direction after the tumbling event. This is reflected in an odd number of sign-changing points for the tumble (see Figure S5 C right panel).
iv. For Case 1, the cells do not change directions before and after the tumble, therefore, all the sign-changing points are removed and it is concluded that no reversal occurred during that event (Figure S5 C left panel).
v. For Case 2, the cells change their directions after the tumble; therefore all the sign changing points in the tumble are treated as one reversal. To assign one of the tumbling points as a reversal point, we identified a point with maximal X coordinates for reversals in which the cell was moving in a positive direction (s=+1) before the reversal. On the other hand, the point with minimal X coordinate was chosen if the cell was moving in a negative direction before the reversal (s=−1). For example, in Figure S5 C right panel, point 5 was assigned as the actual reversal point.

In Figure S5 D, F and G three trajectories are highlighted and the reversal points were labeled with red dots.

The reversal period is defined as the time interval between two adjacent reversal points. If a cell reverses at time *i* and its next reversal occurs at time *j,* then the reversal period τ for the cell is between time *i* and *j.*

$$\tau = (j - i)\delta t \qquad \text{(S.5)}$$

<u>Selection of rippling cells:</u> Under these experimental conditions, even inside the prey region, not all *M. xanthus* cells exhibit rippling behavior. To accurately define this behavior non-rippling cells must be excluded. Therefore, the fraction of cells selected to be tracked as rippling cells was based on the following criteria:
(a) We excluded cells that maintain a low motion state (i.e. cells with an instant speed less than 0.5 μm/min) for longer than 5 min.
(b) We chose the cells for which most movement occurs along the first principal component after rotation of the cell trajectories:

$$\frac{\left|\max(\hat{x}_i) - \min(\hat{x}_i)\right|}{\left|\max(\hat{y}_i) - \min(\hat{y}_i)\right|} > 3 \qquad \text{(S.6)}$$

In Figure S5 D, F and G, three types of cell trajectories are shown: rippling cells on prey (Figure S5 D), non-rippling cells on prey (Figure S5 F) and cells off prey (Figure S5 G).

<u>Estimation of ABM simulation parameters:</u> The parameters for the ABM simulations are summarized in Table S1. Whenever possible the parameters used were estimated directly or indirectly from the experimental data obtained under our conditions. For example, the analysis of individual cell movement described above provides both average cell characteristics (such as average velocity and reversal period) and their population distributions. The agent velocity v used in ABM simulation is the average velocity calculated in the above analysis. The diffusion coefficient D, which is used to characterize the random fluctuation in agent movement, was chosen such that the variance of the instant velocity distribution of the ABM simulation matches the results of the experimental data analysis. Note that the experimentally observed random fluctuations along the x direction and y direction are almost identical. As a result, only one value D is used to represent the noise level in cell movement.

In our ABM simulations of rippling, varying the refractory period changes the average reversal period. Thus, the refractory period was chosen to fit the average reversal period in the ABM to that in the experimental observations of rippling cells. The average reversal period of non-rippling cells observed experimentally was chosen as the natural reversal period T in our ABM and the phase speed ω was calculated using Eq. (5). The diffusion coefficients in the reversal period D_φ were chosen by matching the distribution of reversal periods of the ABM simulations to the experimentally observed distribution. The phase variable φ_0 in the ABM simulations was chosen so that φ_0/ω would equal to the selected refractory period. There are also parameters that cannot be directly estimated experimentally, but can be defined based on the simulation results. For example, the

random noise level D_θ was assigned such that the initially aligned population of cells remains aligned.

Analysis of gray scale images that represent cell densities

As rippling is essentially a traveling cell density wave, a method to acquire cell density information from both the experiments and the ABM simulations was developed. For the experiments, we observed and recorded *M. xanthus* cells preying on *E. coli* cells using fluorescence microscopy at 10X / 20X magnification. The fluorescence intensity in the recorded images represents the local cell density information, so that the lightest areas represent high cell density and the darkest areas represent low cell density. In the ABM simulations, the simulation domain is a 1 µm by 1 µm grid, and the local cell densities are calculated based on the agents whose centers are in each 1 μm^2 grid square. In this analysis, $I(x,y)$, denotes the cell density computed from both our ABM simulation and experimental intensities at position (x,y).

<u>Creation of the cell density space-time images:</u> The cell density space-time plot shown in Figure 2 E was generated from the gray scale DIC images of the experimental cell-density images. We started with a cell-density matrix I of the size X×Y. As most of the cell movements are on the first principal component, we rotated the image I so that the cells mainly move parallel to the X-axis. To rotate image I, we needed a large enough image I', so that it contains all the pixels of image I after rotation. For each pixel $I'(x,y)$ in image I', we computed its corresponding coordinate in image I before rotation:

$$\begin{pmatrix} \hat{x} \\ \hat{y} \end{pmatrix} = (EV^T)^{-1} \begin{pmatrix} x \\ y \end{pmatrix} \tag{S.7}$$

We used the value of the nearest pixel to (x, y) in image I as the pixel value of $I'(\hat{x}, \hat{y})$. If (x, y) was not inside image I, $I'(\hat{x}, \hat{y})$ was set to 0. To obtain the 1-D averaged density I_x, we averaged over the columns of image I':

$$I_x = \left\langle I'(\hat{x}, \hat{y}) \right\rangle_y \tag{S.8}$$

The brackets denote the average over pixel values that were part of the original image. Next, we detrended the I_x by subtracting the least-square fit from itself. As a result, we have the 1-D averaged and detrended cell density of $\hat{I}_x$. By putting the $\hat{I}_x$ of different time-steps together we obtained a 2-D space-time image of cell density. As the last step, we rotated the gray scale image, so that the dark color represents high cell density and bright color represents low cell density. As a result, in the space-time cell density image, the dark ridges represent wave crest (high cell density). We then superimposed the time and position of cell reversals on this plot and the resulting diagram is shown in Figure 2 E. The same procedure was used to produce the space-time plot of the ABM simulation results. The result is shown in Figure 2 F.

We hypothesized that most of the rippling cells reverse during ripple wave crest collisions. To test this hypothesis using the experimental data analysis, the time and positions of cell reversals and the space-time images of cell density were combined. To test this hypothesis using the ABM simulations, the time and positions of cell reversals in the ABM simulations were recorded. The space-time cell density images of both the experimental and ABM simulation data were then processed. As the space-time images are always detrended, the pixels with negative values are discarded (by setting to 0). As a result, the space-time images with only the dark ridges are presented. Next, the locations of the wave crest collisions in the space-time images were determined. Using either experimental or ABM simulation data, each collision area of two wave crests always contains at least one pixel that has the locally maximum cell density since the overlapping of wave crests leads to higher cell density. We identified these local maxima pixels in the space-time images. Subsequently, the rectangular region centered at each pixel with a height equal to double the wave crest width (~20 µm) and a width equal to the time the cells need to cover that width (~3 min) was defined. This represents the collision region of two opposing crests. Next, the time and position of each cell reversal was checked to determine if it falls into the wave collision region. Then, we used a bootstrapping method to calculate the mean and standard deviation of the percentage of reversals inside the wave collision area. The analysis reveals that 75.0% (±2.6%) of all tracked reversals in the prey area occur during wave crests collisions in our experimental observations. In our ABM simulations 82% (±2.1%) of the cells reverse during wave crest collisions. As a control, we added 10% non-rippling cells in the simulations (cells that are not sensitive to signaling), and determined that only 17% (±2.3%) of these cells reverse during wave crests collisions.

Estimation of wavelength and wave crest width:

In both the experimental observations and ABM model simulations, the movement of the *M. xanthus* cells is predominantly the first principal component computed. As a result, the direction of ripple movement is also the first principal component. Thus, the 1-D continuous wavelet transform (CWT) was used to obtain the wavelength [1-4]. Given a function (or signal) *f(x)*, a mother wavelet $\psi(x)$, the CWT is defined as:

$$c(a,b) = \int_{-\infty}^{+\infty} f(x) \frac{1}{\sqrt{a}} \psi^{*}\left(\frac{x-b}{a}\right) dx \tag{S.9}$$

where a is the scale parameter and $a > 0$; b is the position parameter; $c(a,b)$ denotes the wavelet coefficient computed from Eq. (S.9) at scale a and position b; * denotes the complex conjugation.

Above, the original experimental images were rotated, such that the first principal component of the images is parallel to the X-axis. In the ABM simulations, agents aligned along the X-axis initially and the alignment rule keeps the agents aligned during the simulation. As a result, for both types of images, the direction of ripples is along the

X-axis. If in image *I* with size $X \times Y$ ripples travel along the X-axis, then the CWT is applied to each row of the image: $I_i(x) = I(:,i)$, where *i = 1, 2,…,Y*. To apply the CWT to $I_i(x)$, would assume that $I_i(x)$ is the result of sampling of a continuous function.

There are many mother wavelet functions, among which we chose the Morlet wavelet [5,6]:

$$\psi(x) = e^{-x^2/2}\cos(5x) \qquad \text{(S.10)}$$

As a result, for each $I_i(x)$ there is a corresponding $c_i(a,b)$ using equation (S.9). The next step is to average the wavelet coefficient along all *i* (along y-direction).

$$\tilde{c}(a,b) = \frac{1}{Y}\sum_{i=1}^{Y} c_i(a,b) \qquad \text{(S.11)}$$

In Figure S3 A and C, several peaks in the absolute value of wavelet coefficient are observed. Wavelengths are calculated fom the distances along the X-axis (horizontally) between every other peak in the absolute value of wavelet coefficients. In order to do that the average $\tilde{c}(a,b)$ over the scale parameter *a* was determined:

$$\tilde{c}(b) = \frac{1}{N_0}\sum_{a=1}^{N_0} \tilde{c}(a,b) \qquad \text{(S.12)}$$

Where N_0 is the largest scale used in the wavelet transform. If in $\tilde{c}(b)$, there are *N* peaks with position parameter $b_1,\ldots,b_N$, the average wavelength λ of one image is calculated by:

$$\lambda = \frac{\sum_{i=1}^{N-2}(b_{i+2} - b_i)}{N-2} \qquad \text{(S.13)}$$

Eq. (S.13) is used for the ABM simulation images. For the experimental images, the equation is slightly changed to omit the peaks at the two ends b_1 and b_N. This is because the peaks at b_1 and b_N are deeply affected by the discontinuity at the edges of the DIC images. The periodic boundary condition for the ABM simulation was used, so there is no effect of discontinuity at the edges. Then the average wavelength λ from different frames was used to obtain the mean wavelength (solid line in Figure 2 A and B) and the standard deviation (error bar in Figure 2 A and B). Twenty experimental images and 20 ABM simulation images were used to calculate these data points.

To determine the wave-crest width, we used a similar procedure, but assumed that the wave crest is well approximated by a Gaussian distribution

$$N(x,\sigma) = e^{-\frac{x^2}{2\sigma^2}} \qquad \text{(S.14)}$$

Therefore, the best-fit Gaussian distribution was obtained and its width σ was used as our estimate of the wave crest. Starting with 1-D densities $I_i(x)$, the moving correlation coefficients of the Gaussian function and $I_i(x)$ were calculated:

$$f_i(\sigma, b) = \int_0^X I_i(x) \frac{1}{\sigma} N(x-b, \sigma) dx \quad \text{(S.15)}$$

Then, the average of the correlation coefficients was determined:

$$\tilde{f}(\sigma) = \frac{1}{XY} \sum_{b=1}^{X} \sum_{i=1}^{Y} f_i(\sigma, b), b) \quad \text{(S.16)}$$

and the wave crest width Δ was obtained by:

$$\Delta = \arg\max_{\sigma} \tilde{f}(\sigma) \quad \text{(S.17)}$$

Bifurcation diagram for the signaling probability

Using the wavelet coefficients calculated in the previous step, an order parameter that describes the existence of the waves was derived. If the averaged 1-D wavelet coefficients calculated using equation (S.12) of an image with no wave is $\tilde{c}_0(a)$, and there are some wavelet coefficients $\tilde{c}(a)$ using the same equation, then the order parameter OP is defined as:

$$OP = \max(\tilde{c}(a) - \tilde{c}_0(a)) \quad \text{(S.18)}$$

For simulations with $OP < 0.4$, no visible ripple pattern was observed, but when $OP > 0.4$, a ripple pattern was observed. By gradually decreasing the signaling probability we discovered that waves disappeared when the signaling probability reached a critical value. It was concluded that the signaling probability serves as a bifurcation parameter in the ABM simulations for ripples. The OP was used for the quantification of the ripple pattern emergence and Figure S3 E illustrates the conclusion: the order parameter OP is ultrasensitive to increases in the signaling probability around the threshold value of 0.05 above which ripples appear.

Estimating colony expansion in experimental images

ImageJ software was used to obtain *M. xanthus* colony edge information from analysis of the gray scale images from the DIC microscopic experimental observations. For each image, we manually picked points on the colony edge and recorded the positions. The number of points was chosen to be large enough so that the linear interpolation of these points can sufficiently represent the real colony edge. Then, we used linear interpolation to connect these points and as a result, we obtained a curve that represents the colony edge. To calculate the movement of the edge between images, the following procedure was applied:

(i) Two curves of the edges of the same colony from two different times; curve 1 and curve 2 were examined. For each line segment in curve 1, we computed the position of the mid-point of the line segment by averaging the coordinates of the two end points.
(ii) The distance from the mid-point of each of the line segment in curve 1 to curve 2 was then computed and recorded.
(iii) The movement of the colony from one image to the other was defined as the average distance of the distances calculated in (ii) above.

References:

1. Morlet J, Arens G, Fourgeau E, Giard D (1982) Wave propagation and sampling theory---Part II: Sampling theory and complex waves. Geophysics 47: 222-236.
2. Mallat SG (1989) Multiresolution approximations and wavelet orthonormal Bases of L2(R). Transactions of the American Mathematical Society 315: 69-87b
3. Mallat SG (1989) A theory for multiresolution signal decomposition: the wavelet representation. IEEE Trans Pattern Anal Mach Intell 11: 674-693.
4. Werner R, Stebel K, Hansen GH, Blum U, Hoppe UP, et al. (2007) Application of wavelet transformation to determine wavelengths and phase velocities of gravity waves observed by lidar measurements. Journal of Atmospheric and Solar-Terrestrial Physics 69: 2249-2256.
5. Daubechies I (1990) The wavelet transform, time-frequency localization and signal analysis. Information Theory, IEEE Transactions on 36: 961-1005.
6. Jeremy FA, James SW (2002) Time-frequency analysis of musical instruments. SIAM Rev 44: 457-476.
